# Competing Colloidal Interactions Govern Polymer Encapsulation of Hydrophobic Iron Oxide Nanoparticles

**DOI:** 10.1021/acs.langmuir.6c04393

**Published:** 2026-10-08

**Authors:** Soumodeep Biswas, Takreem A. Sindhu, Muhammad Salman Haider, Ethayaraja Mani, Sulalit Bandyopadhyay

**Affiliations:** † Department of Chemical Engineering, 37268 Indian Institute of Technology Madras, Chennai 600036, India; ‡ Particle Engineering Center, Department of Chemical Engineering, 66959 Norwegian University of Science and Technology, Trondheim 7491, Norway; § School of Chemical and Materials Engineering, 529979 National University of Science and Technology, Islamabad 44000, Pakistan; ∥ Centre of Excellence in Biodegradable Packaging, Department of Chemical Engineering, Indian Institute of Technology Madras, Chennai 600036, India; ⊥ Centre for Soft and Biological Matter, 8018 Indian Institute of Technology Madras, Chennai 600036, India; # School of Sustainability, Indian Institute of Technology Madras, Chennai 600036, India

## Abstract

Control over synthesis conditions is necessary to ensure high loading in polymer-encapsulated iron oxide nanoparticles (P-IONPs) and enable biomedical applications. While flash nanoprecipitation (FNP) is a robust method to fabricate P-IONPs, very little is known regarding the response of hydrophobic IONPs to the change in solvent quality during FNP and its effect on the nature of encapsulation. In this work, we first probe the sizes of oleic acid (OA)-coated IONPs at varying compositions of water (ϕ_w_) in a tetrahydrofuran (THF)–water mixture through time-based dynamic light scattering (DLS) and cryo-electron microscopy. It was found that OA-IONPs existed as large (micron)-sized clusters in THF, which broke into smaller clusters as ϕ_w_ in the solvent mixture increased. A free energy model of clustering is presented to describe the competing repulsive and attractive interparticle interactions driving the equilibrium cluster sizes (d*) as a function of ϕ_w_. This effect was further pronounced when an aqueous Tween 80 solution was added to the dispersion, resulting in individually dispersed IONPs at high ϕ_w_. The d* directly influenced the nature of P-IONPs produced using FNP at varying aqueous-to-organic flow rate ratios. Finally, a systematic study was performed to investigate the effects of the concentration of polymer and IONPs, and OA surface coverage on IONPs, on the extent and nature of encapsulation. The findings suggest the key role of competing colloidal interactions in governing the fate of synthesized P-IONPs via FNP. The understanding from this study would enable the rational design of P-IONPs with high, uniform loading for desired applications.

## Graphical abstract

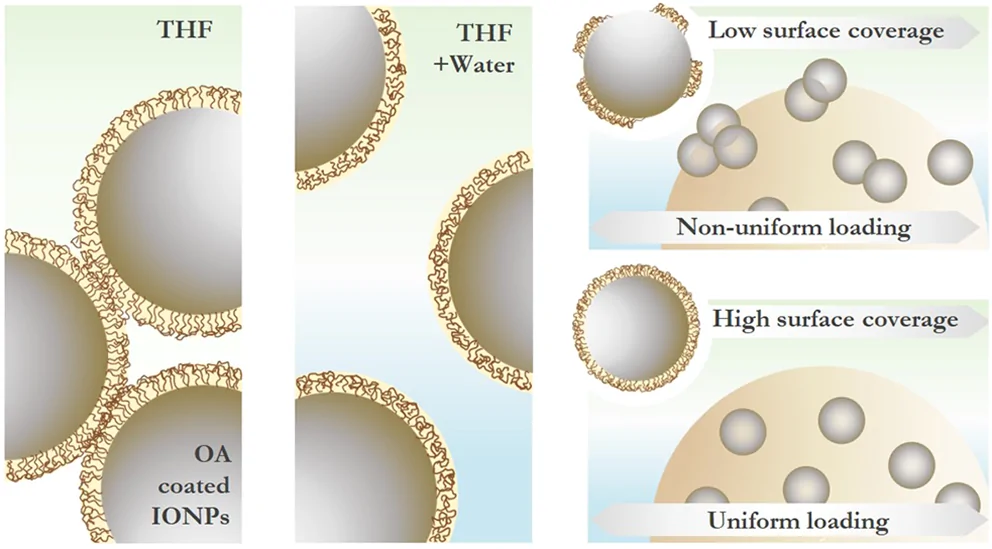

## Introduction

Polymer-encapsulated hydrophobic cores, such as molecular dyes, drugs, and metallic particles, are of great interest for biomedical applications.^1–3^ These polymer-encapsulated cores are potential candidates for imaging contrast agents,^4^ carriers for targeted delivery,^5^ and theranostic platforms that combine imaging and therapy.^6^ Polymer-encapsulated iron oxide nanoparticles (P-IONPs) have emerged as both model and application systems for study.^7,8^ The magnetic properties of IONPs enable their use in magnetic resonance imaging (MRI), magnetic hyperthermia, and magnetically guided drug delivery.^9,10^ Therefore, IONPs are widely identified as superior nanocarriers due to their ability to simultaneously perform diagnostic and therapeutic functions.^11,12^ Polymer encapsulation of IONPs provides enhanced colloidal stability and biocompatibility to enable *in vivo* applications.^13,14^ Recently, we have shown that paclitaxal-loaded P-IONPs could be used for alternating magnetic field-guided drug release.^15^ The enhanced drug release under a magnetic field, combined with biocompatibility evaluations, ensures P-IONPs as potential carriers for magnetically guided therapeutics.

The synthesis of P-IONPs has been reported through various routes. For example, Nguyen et al. reported the uniform coating of IONPs with various dye polymers using free-radical emulsion polymerization mediated by macro-RAFT copolymer stabilizers.^16^ IONPs coated with dendrimers and polymers, including dextran, poly­(ethylene glycol) (PEG), and poly­(lactic-*co*-glycolic acid) (PLGA), through grafting have also been widely reported.^17^ These grafting methods exhibit some limitations, including toxic residual contaminants, complex multistep synthesis procedures, poor reproducibility, limited drug-loading efficiency, and stability concerns under physiological conditions.^18^ Although the conventional ways of coating IONPs with polymers have been widely studied, lately, flash nanoprecipitation (FNP) was proposed as a straightforward and scalable alternative to the above techniques for encapsulating hydrophobic as well as hydrophilic cores.^19,20^ Typically, in an FNP process, the dissolved polymers in organic media along with the dispersed hydrophobic particles are rapidly mixed with an aqueous stream in a micromixer to precipitate polymer-encapsulated nanoparticles. This technique has been widely reported for producing various encapsulated hydrophobic cores, including drugs and preformed metal nanoparticles.^21^

Through the course of the development, formation mechanisms of polymer-encapsulated nanoparticles via the FNP process have been of great interest for study to enable ratonal design of experiments. A mechanistic explanation of the encapsulation of hydrophobic cores by block-*co*-polymers (BCPs) was reported by Pagels et al.^3^ The authors derived a scaling law for the final particle size based on Smolochowski’s aggregation model, assuming that the aggregation of the hydrophobic core molecules was followed by that of BCP chains. An empirically fitted constant explains the experimentally observed particle sizes across various core-BCP systems. More lately, Wilson and Prud’homme^8^ reported the coencapsulation of hydrophobic polystyrene particles with IONPs as model systems inside a block-*co*-polymer shell using FNP.^8^ In contrast to the sequential aggregation model proposed in ref 3, the authors discuss the significance of competing time scales of the core-diffusion and polymer precipitation, which govern the encapsulation process. Through our previous studies,^15,22^ we have systematically shown that the surface properties combined with the size of IONPs play a crucial role in determining the fate of their encapsulation. Although there have been attempts in understanding the factors governing the encapsulation of hydrophobic entities by polymers via FNP, the aim has been optimizing the output metrics.

The colloidal stability of IONPs with various surface functionalizations in single-solvent systems has been previously investigated and reported.^23–25^ However, the role of colloidal behavior of IONPs in a mixed-solvent environment during the course of encapsulation in governing the fate of encapsulation has been seldom probed. Therefore, we address the following questions in this work: (i) How do hydrophobic IONPs arrange themselves in a mixed (aqueous–organic)-solvent system during the course of encapsulation? (ii) How do these assemblies of IONPs at a given aqueous to organic composition influence the nature of their encapsulation via FNP? (iii) How does surface functionalization and its extent affect the loading and distribution of encapsulated IONPs? We address these questions by first synthesizing hydrophobic oleic acid-coated IONPs by two different methods—thermal decomposition in octadecane and eicosane and coprecipitation—resulting in three different sizes (11, 15, and 26 nm). All three categories of IONPs were then dispersed in tetrahydrofuran (THF). The excess unbound oleic was then removed through multiple wash cycles until the oleic acid amounts in the IONP dispersion did not change significantly. We then probe the colloidal behavior of IONPs in varying compositions of mixed-solvent systems by kinetically monitoring hydrodynamic sizes using dynamic light scattering. The trend of IONP cluster sizes with the composition of mixed solvent was explained using a colloid clustering model based on an appropriate description of interparticle interaction energies. The effect of IONP-cluster sizes determined by the flow rate ratio of aqueous and organic streams in FNP was tested for PLGA encapsulation of IONPs in order to visualize the nature of encapsulation under these conditions. Finally, we systematically investigated the three categories of IONPs at two concentration levels of both PLGA and IONPs to evaluate encapsulation efficiency across the systems and the distribution of IONPs inside the PLGA particle matrix. The nature of encapsulation was then explained using hydrophobicity and size of IONPs, which drive the competing interactions governing the fate of their encapsulation.

## Experimental Section

### Materials

For the iron oxide synthesis, octadecane (ODE) (CAS 593-45-3) and eicosane (ES) (CAS 112-95-8) were obtained from Evonik. Sodium oleate (CAS 143-19-1), oleic acid (OA) (CAS 112-80-1), hexane (CAS 110-82-7), isopropanol (CAS 67-63-0), acetone (CAS 67-64-1), tetrahydrofuran (THF) (CAS 109-99-9), ferrous chloride (FeCl_2_·4H_2_O) (CAS 13478-10-9), ferric chloride (FeCl_3_·6H_2_O) (CAS 10025-77-1), ethanol (CAS 1404-93-9), and 25% ammonium hydroxide (CAS 1336-21-6) were obtained from Sigma-Aldrich. Tween 80 (polysorbate 80) (CAS 1404-93-9), 65% nitric acid, and phosphotungstic acid (PTA) stain used in FNP and imaging were also obtained from Sigma-Aldrich. PLGA Resomer 502 and Resomer 503 were obtained from Evonik. Capsules of size 0 and 0.68 mL capacity made of gelatin, used for the digestion of NPs, were obtained from Capsuline.

## Methods

### Synthesis of IONPs

Three different categories of IONPs were synthesized for this study using thermal decomposition and coprecipitation methods. Two of these categories of IONPs were synthesized using thermal decomposition in octadecane and eicosane solvents and are termed ODE- and ES-IONPs, respectively. The third category of IONPs was synthesized by the coprecipitation method and is termed CP-IONPs. The details of the syntheses are given as follows.

#### Thermal Decomposition

Iron oleate was synthesized as a precursor for the preparation of IONPs using thermal decomposition following our previous work.^22^ Briefly, a 250 mL round-bottom flask, 25 mL of Milli-Q (MQ) water, 70 mL of hexane, and 40 mL of ethanol were added. The flask was placed in an oil bath and stirred at 1000 rpm. 5 g of FeCl_3_.6H_2_O and 18.25 g of sodium oleate were added to the solvent mixture. The reaction mixture was then maintained at 70 °C for 4 h under continuous stirring. After completion, the mixture was cooled to room temperature and washed three times with MQ water to remove unreacted species. Iron oleate was then recovered using a rotary evaporator (RE). The water bath in the RE was set to 40 °C and the rotation was set to 200 rpm. Hexane and water were removed from the product by operating the RE at variable pressures ranging from 200 mbar to 40 mbar, until a dark viscous brown liquid was obtained. The final product was then stored in a glass vial at 4 °C for subsequent use in the synthesis of IONPs via thermal decomposition.

Two different categories of IONPs were synthesized via thermal decomposition, differing in the high-boiling solvent used, namely ODE and ES, as previously reported.^22^ OA and iron oleate were premelted prior to use by gently heating them in a warm water bath. In a 100 mL round-bottom flask, 600 μL of OA and 1.6 g of iron oleate were mixed with 25 mL of either ODE or ES, depending on the desired synthesis route. The reaction mixture was stirred at 300 rpm and heated under controlled conditions based on previous reports.^26,27^ For ODE-IONP synthesis, a heating ramp rate of 3 °C min^–1^ was applied, followed by a dwell time of 45 min at 318 °C. ES-IONPs were synthesized at an elevated ramp rate of 6 °C min^–1^ with a dwell time of 30 min at the same reaction temperature (318 °C). Upon completion of the reaction, the mixture was allowed to cool, after which 25 mL of hexane was added. The resulting solution was then poured into a beaker containing 50 mL of isopropanol and 50 mL of acetone to induce IONP precipitation. The suspension was placed on a magnetic separator for 10 min to collect the IONPs, and the supernatant was discarded. The IONPs were washed three times with acetone while subjecting to magnetic separation and finally redispersed in 30 mL of THF.

#### Coprecipitation

The third category of IONPs was synthesized via the coprecipitation method. In this method, 84.6 g of MQ water was placed into each of four separate beakers, and 15.4 mL of 25% ammonium hydroxide (NH_4_OH) was added to each beaker along with a magnetic stir bar. Separately, an iron salt solution was prepared by dissolving 4 g of FeCl_2_·4H_2_O and 10.8 g of FeCl_3_·6H_2_O in MQ water in a 50 mL conical flask. This iron chloride solution was transferred to a buret for controlled addition. The iron salt solution was then added dropwise to the NH_4_OH solution at a controlled rate of approximately three drops per second (10 mL of iron chloride solution per 15.4 mL of NH_4_OH solution) under continuous stirring. Immediately after the completion of the addition, the magnetic stirring bar was removed and the beaker was placed on a magnet to facilitate the separation of IONPs. The supernatant was discarded, and the NPs were washed three times with MQ water. Finally, the purified IONPs were redispersed in 25 mL of MQ water for further use.

#### Phase Transfer of Hydrophilic Coprecipitated IONPs

Hydrophilic IONPs, obtained using coprecipitation, were rendered hydrophobic via surface functionalization with OA through a phase transfer process. The functionalization was carried out in a temperature-controlled ultrasonic bath maintained at 67 °C. In a screw-capped vial, premelted oleic acid was added to a dispersion of coprecipitated IONPs at 1.2 mL per 200 mg dried IONP mass. The resulting suspension was sonicated for 2 h to allow adsorption of OA onto the IONP surface. Following sonication, IONPs were magnetically separated and washed three times with a 1:1 (v/v) ethanol–water mixture to remove excess, unbound OA. The oleic acid-coated, hydrophobic IONPs were subsequently redispersed in THF for further characterization and use.

### Purification and Removal of Excess Oleic Acid

Hydrophobic IONPs may contain both surface-bound OA and free OA in the dispersion. The free OA was removed through multiple wash cycles and the intermediate quantification of OA. To determine the number of washing cycles required for the effective removal of free OA, all three types of synthesized IONPs were subjected to systematic washing studies using a 1:1 acetone:isopropanol solvent mixture. For this analysis, 3 mL aliquots of IONPs synthesized via thermal decomposition were transferred to four separate screw-capped vials. 5 mL of a washing solvent mixture was added to vials containing IONPs, followed by vortexing and sonication for 30 s. Each of these four vials were subjected to varying numbers of wash cycles, 3, 6, 9, and 12. After washing, the IONPs were magnetically separated and dried in Eppendorf tubes and then transferred to gelatin capsules for Speedwave digestion. The iron content in the samples was quantified using microwave plasma atomic emission spectroscopy (MP-AES), which was further used to calculate the organic content in the sample. The schematic for OA removal is presented in the SI (see Figure S3).

### Polymer Encapsulation of IONPs Using FNP

A modified FNP method was carried out for the encapsulation of IONPs in a poly­(lactic-*co*-glycolic acid) (PLGA) matrix using a multi-inlet vortex mixer (MIVM) with two inlets and LAMBDA VIT-FIT syringe pumps set to a driving force of 300 N. The organic stream consisted of THF containing dissolved PLGA and dispersed IONPs, loaded into a 20 mL polypropylene syringe. The aqueous (antisolvent) stream comprised 0.1% (w/v) Tween 80 solution in water, loaded into a 120 mL polypropylene syringe. The syringes were connected to the MIVM using appropriate tubing and ferrules, and the experimental setup was assembled as illustrated in Figure 1c. Following sample collection, magnetic separation was carried out by placing the centrifuge tubes against a permanent magnet for 30 s, after which the supernatant mostly containing uncoated IONPs was discarded. The residue was centrifuged at 8000 rpm for 5 min to obtain PLGA-encapsulated IONPs (P-IONPs). The pelleted IONPs were further subjected to two cycles of washing by dispersing in water followed by centrifugation and finally redispersed in water for further characterization.

**Figure 1:**
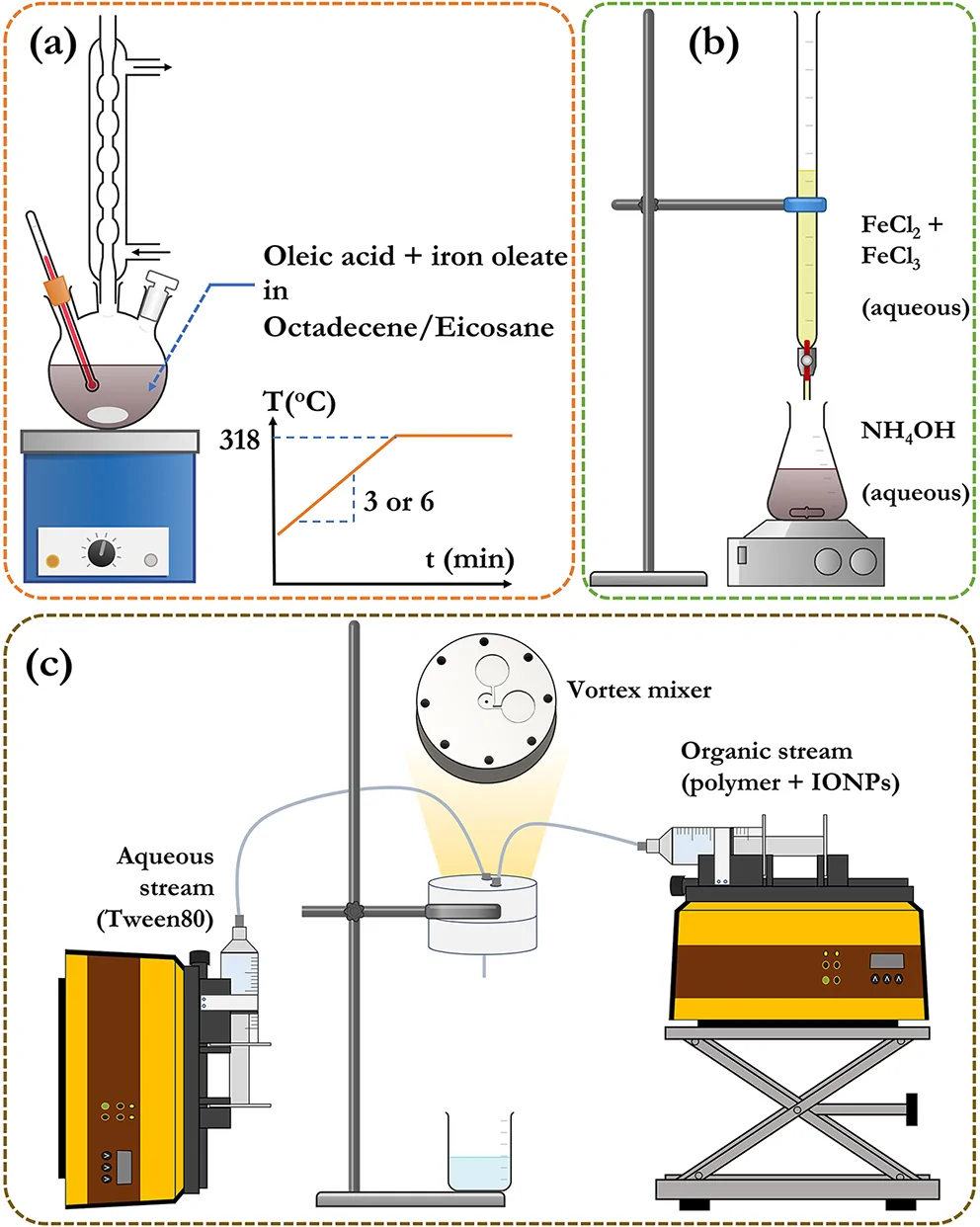
Schematics of experimental setups used for synthesis and encapsulation of IONPs: (a) Thermal decomposition method for the synthesis of oleic acid-functionalized IONPs in either octadecane or eicosane. A temperature ramp is provided to heat the reaction mixture to 318 °C. (b) Coprecipitation method for the synthesis of IONPs where the precursor solution containing iron salts is added dropwise to a bath of reducing agent, ammonium hydroxide (NH_4_OH), to form IONPs. (c) Flash nanoprecipitation setup for the synthesis of polymer-encapsulated IONPs injecting the organic stream (polymer dissolved and IONPs dispersed in THF) and aqueous stream (aqueous Tween 80 solution) into a multi-inlet vortex mixer (MIVM).

### Characterization of IONPs and P-IONPs

#### Quantification of the Total Iron and Organic Content

Microwave plasma atomic emission spectroscopy (Agilent 4210-MPAES) was used for the elemental quantification of iron in a given sample of IONPs and P-IONPs. Prior to elemental quantification, the samples were subjected to microwave-assisted acid digestion using a Speedwave XPERT microwave pressure digestion system to convert them into their elemental form. The dried samples in digestion capsules were placed into digestion vessels containing 10 mL of 65% (v/v) nitric acid solution. The digestion procedure was carried out in two stages: the temperature was first increased to 180 °C at a ramp rate of 15 °C min^–1^ and maintained for 15 min, followed by a second stage in which the temperature was raised to 210 °C and held for 30 min. Upon completion, the resulting solutions were transferred to centrifuge tubes and diluted to the required volume with Milli-Q water prior to analysis.

MP-AES determines the elemental concentration in a sample by measuring its emission intensity and comparing it to the calibration standards of known concentrations. The analyte concentration was calculated using a calibration curve constructed from these standards, provided in the SI (see Figures S1 and S2). The digestate collected from Speedwave digestion was analyzed in MP-AES at suitable dilutions guided by the calibration curve. Details of the estimation of oleic acid mass and encapsulation efficiency are provided in the SI.

#### Electron Microscopy

The synthesized IONPs and P-IONPs were imaged using a Hitachi S-5500 scanning (transmission) electron microscope, S­(T)­EM. The imaging was performed at an accelerating voltage in the range of 20–30 kV and an emission current of 15–25 μA. The samples of P-IONPs were prepared for electron microscopy on TEM grids using negative staining. 1.5% (w/v) phosphotungstic acid (PTA) solution in 0.01 M HEPES buffer prepared with Milli-Q water was used as the staining agent, and the pH of the stain was adjusted to 6.5. An aliquot of 10 μL of the undiluted PIONP suspension was deposited onto a TEM grid and allowed to dry for 2 h. Subsequently, 10 μL of the PTA stain was cast onto the grid with dried P-IONPs and left for 2 min and carefully blotted using Kimtech wipes. The S­(T)­EM micrographs were analyzed using the image analysis tool ImageJ^28^ to obtain particle size distributions, by measuring sizes of at least 300 particles per sample.

The colloidal assemblies of hydrophobic iron oxide nanoparticles in solvent and solvent–aqueous mixtures were imaged under a Talos F200i cryo-electron microscope (TFS) operating at an accelerating voltage of 200 kV. The samples for cryo-electron microscopy (cryo-EM) were prepared by vitrification using a Vitrobot Mark IV system (TFS). Cu300 R1.2/1.3-type (Quantifoil GmbH) grids were glow-discharged at 25 mA current for 60 s duration (GloQube Plus, U.K.) prior to vitrification. Briefly, 3 μL of the sample was applied to the carbon support side of the glow-discharged grid, which was kept inside the blotting chamber controlled at 22 °C and 100% relative humidity. The grid was then blotted for 3 s to remove the excess sample and immediately plunged into liquid ethane that was cooled by liquid nitrogen. Subsequently, the grid was mounted onto a cryo-holder (ELSA model 698, GATAN USA) kept at liquid nitrogen temperature for imaging. The images were taken in low-dose mode with the exposure time being varied from 3 to 6 s using a Ceta 16 M camera (TFS).

#### Time-Dependent Size Measurements Using Dynamic Light Scattering

Time-dependent measurements for hydrodynamic sizes of IONPs in mixed aqueous and THF solvent mixture systems were performed using Anton Paar Litesizer 500 dynamic light scattering equipment. The particle concentration was kept constant at ≈10^10^ particles per mL in the total mixture, for all the systems. The number concentration in the stocks was estimated based on the total iron content, determined by elemental analysis, and the average size of IONPs, determined by image analysis of the STEM micrographs. A user-defined setting of 5 process runs of 10 s each with no equilibration time and the backscattered detector at 175° was set in the ”series size measurement” mode in particle analysis software Kalliope, provided with the equipment. The results of the repeated measurements were then converted to kinetic data using the time stamps provided for the start of each measurement.

## Results and Discussion

### Synthesis of IONPs Using Thermal Decomposition and Coprecipitation

The IONPs synthesized by the methods discussed in the Synthesis of IONPs were imaged under S­(T)­EM. Representative S­(T)­EM images of the synthesized ODE-, ES-, and CP-IONPs are presented in Figure 2a–c, respectively. The sizes of the particles were obtained by image analyses of the S­(T)­EM micrographs. The histograms corresponding to the sizes are provided in the inset of each S­(T)­EM micrograph. The sizes of ES- and OCE-IONPs were estimated to be 26 ± 5 nm and 11 ± 2 nm, respectively. The effect of the solvent used for the synthesis of IONPs through the thermal decomposition method on the sizes obtained has been previously reported by our group.^22^ These findings are consistent with the reported mechanism, which suggest that higher boiling solvents result in larger particles due to enhanced growth at elevated temperatures.^29,30^ Therefore, the ES-IONPs were larger in size. The sizes of CP-IONPs were estimated to be 15 ± 2 nm.

**Figure 2:**
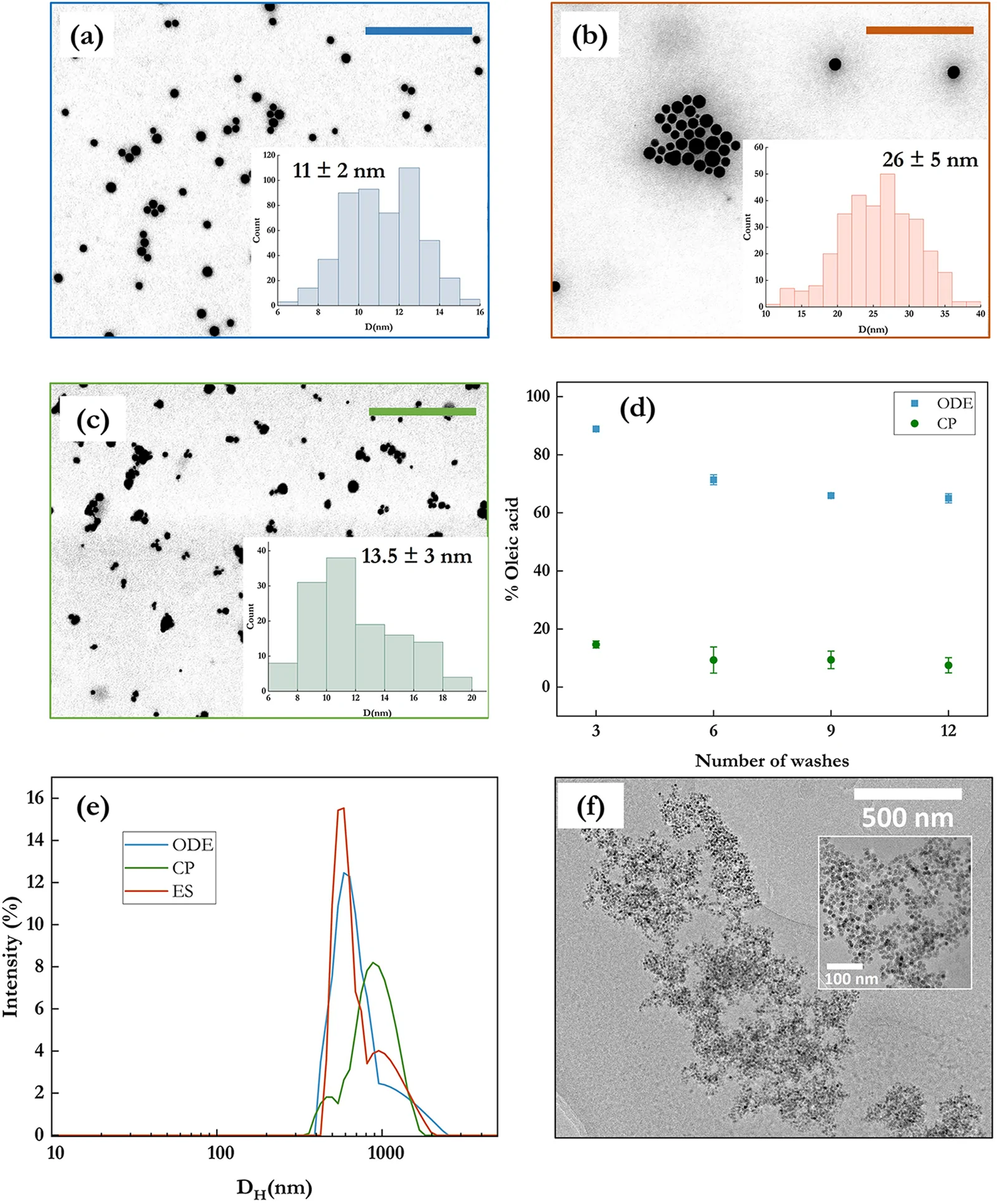
S­(T)­EM micrographs of IONPs synthesized using thermal decomposition with (a) octadecane (ODE) and (b) eicosane (ES) as solvents during synthesis and (c) coprecipitation (CP) method. The insets in (a), (b), and (c) correspond to the histograms obtained from image analysis of the particle sizes. Scale bars are 200 nm. (d) Quantified OA content plotted against the number of wash cycles for the ODE and CP classes of IONPs. (e) Size distributions for ODE-, ES-, and CP-IONPs when dispersed in THF, obtained using DLS. (f) Cryo-EM micrograph of ODE-IONPs suspended in THF. The inset shows a magnified view of the IONP-assemblies in THF.

Thermal decomposition produces OA-coated hydrophobic IONPs, with the functionalization taking place during the course of synthesis. In contrast, the coprecipitation method produces hydrophilic IONPS, which, in this work, were phase-transferred to organic media by an *ex situ* postsynthesis functionalization with OA. Therefore, we expect different extents of OA coating and eventual free (unbound) OA among the different categories of IONPs. In order to study the colloidal behavior of these three categories of IONPs, it is therefore necessary to remove the excess (unbound) OA following a washing procedure as described in the Purification and Removal of Excess Oleic Acid section. The aliquots of IONPs subjected to certain wash cycles were characterized using MP-AES to quantify the amount of OA. Figure 2d shows the plot for the percentage of OA present in CP- and ODE-IONPs against the number of wash cycles applied. The results show that for ODE-IONPs, nine wash cycles were needed to effectively remove all free OA as the amount of OA did not change significantly after three more wash cycles. However, six wash cycles were enough to remove free OA from CP-IONPs, indicating loosely bound coating as a result of *ex-situ* functionalization. Subsequently, ES-IONPs were washed for nine cycles to remove unbound OA, similar to ODE-IONPs, as they were synthesized using the same method.

Following the OA removal, we probed the sizes of IONPs in THF using dynamic light scattering (DLS). The DLS measurements revealed that all three categories of IONPS, ODE, CP, and ES, exist as large clusters in the THF. Figure 2e shows the size distributions of ODE, CP, and ES categories of IONPs, obtained using DLS. The DLS measurements were performed in triplicates. In THF, the ODE-, ES-, and CP-IONPs exhibited average hydrodynamic diameters (*D*_H_) of 945, 1609, and 1910 nm, with polydispersity indices (PDIs) of 0.212, 0.72, and 0.382, respectively. The size distributions suggest that all three classes of IONPs exist as micron-sized clusters when dispersed in THF. To confirm these observations, we imaged ODE-IONPs under cryo-EM to capture the state of IONPs in the solvent. The vitrification process preserved the native states of the particle assemblies and kinetically trapped solvent molecules in a disordered glassy state as a result of rapid cooling.^31^ Figure 2f shows a representative micrograph of ODE-IONPs dispersed in THF obtained using cryo-EM. The IONPs could be seen tightly aggregated as clusters when dispersed in THF, captured in the frozen solvent. Supporting cryo-EM images are provided in the SI (see Figure S5). These images confirmed that IONPs indeed exist as large micron-sized clusters when dispersed in THF. Since THF is more polar than other nonpolar solvents with a dielectric constant of ≈7.5,^32^ the hydrophobic chains of OA interact with each other. The hydrophobic interactions between OA chains on neighboring particle surfaces, combined with the magnetic and van der Waal’s attractive interactions, possibly result in the formation of large IONP clusters.^24^

### Effect of Water Addition on IONP Sizes

In order to understand the colloidal behavior of hydrophobic IONPs during the polymer encapsulation process using FNP, we investigated the effect of water addition on the sizes of IONPs in a controlled manner. Water was added to a dispersion of IONPs in THF at a predefined ratio while maintaining constant particle concentration. Time-dependent DLS measurements were performed in order to monitor the sizes of IONPs from the instance of water addition. Figure 3a shows the results from these measurements, where the hydrodynamic diameter (*D*_H_) is plotted against the time (*t*) passed since the addition of water. The time delay between the addition of water to the IONP-THF mixture and the first measurement was logged for each system and added while plotting the time-dependent sizes of IONPs. The shaded area represents the standard deviation of the distribution corresponding to each measurement, obtained by multiplying the mean size with the coefficient of variation reported by particle analysis software Kalliope. The mixing ratio (*X*:*Y*) in the legends refers to the aqueous (*X*) to organic (*Y*) flow rates (mL min^–1^) corresponding to FNP. For the sake of discussion, we will refer to the mixing ratio in terms of volume-based aqueous composition in the THF–water mixture (solvent mixture). There are two important observations to be made in Figure 3a—(1) there exists an effective equilibrium finite cluster size at a given aqueous composition (ϕ_w_) and (2) the equilibrium cluster sizes decrease at higher water fractions: ≈570 nm at ϕ_w_ = 0.5 to ≈60 nm at ϕ_w_ = 0.91. We attempted to visually verify the effect of addition of water on the IONP clusters. Figure 3b shows the micrograph of IONP clusters at ϕ_w_ = 0.91, obtained using cryo-EM. We found that although IONPs formed seemingly large clusters of around 500 nm, these clusters were made of many loosely bound smaller clusters. The inset in Figure 3b shows small clusters of sizes in the range of 100–200 nm, supporting the observations made using DLS. Therefore, when compared to the tightly packed IONP clusters formed in pure THF (shown in Figure 2e), the DLS measurements and the cryo-EM micrographs indicate that the addition of water results in breaking of these clusters and loosening of particle packing. Supporting cryo-EM micrographs corresponding to ϕ_w_ = 0.91 are presented in the SI (see Figure S6).

**Figure 3:**
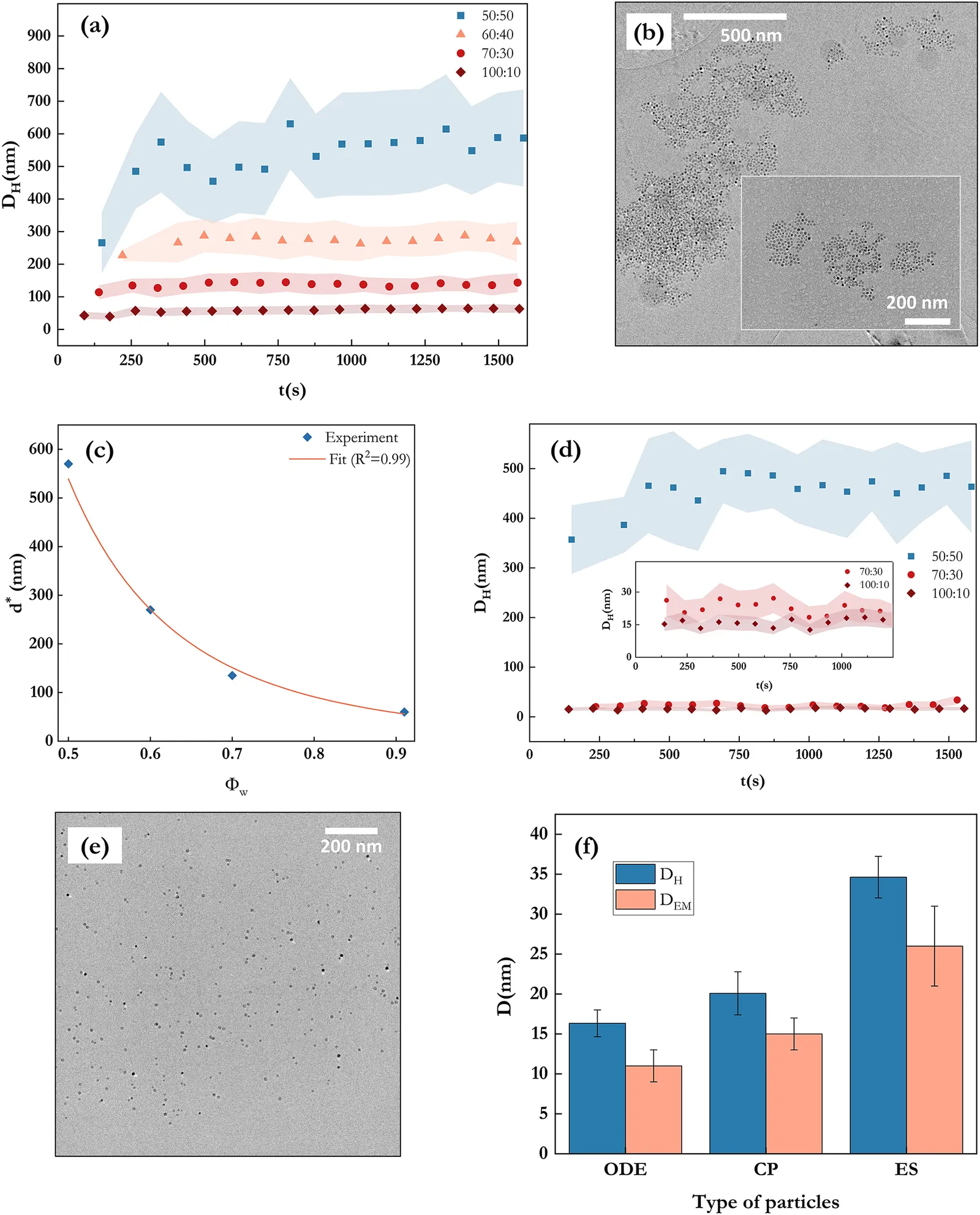
(a) Time-dependent hydrodynamic size (*D*_H_) measurements of IONPs at varying compositions of water, indicated in the legend as *X*:*Y*, corresponding to the mixing rates of aqueous (*X*) and organic (*Y*) streams in FNP. (b) Cryo-EM micrograph of ODE-IONPs suspended in the THF–water mixture at a ϕ_w_ of 0.91. (c) Mean *D*_H_ of ODE-IONPs suspended in the THF–water mixture plotted against the corresponding ϕ_w_. The continuous line corresponds to the fitted curve given by eq 6. (d) Kinetic *D*_H_ measurements of IONPs at varying compositions of 0.1% aqueous Tween 80 (T80) solution in the THF-T80 mixture. The inset represents a zoomed-in plot for *D*_H_ at 70:30 and 100:10 compositions. (e) Cryo-EM micrograph of ODE-IONPs suspended in the THF-T80 mixture at a T80 composition of 0.91. (f) Comparison of IONP sizes measured using DLS (*D*_H_) at a T80 solution composition of 0.91 and electron microscopy images (*D*_EM_).

We propose a mechanism for the formation of finite size clusters of OA-coated IONPs at a given aqueous composition based on a competing attractive and repulsive interparticle interactions. We describe the free energy for a case of two-dimensional (2D) colloidal cluster formation using a short-range attraction long-range repulsion (SALR) potential, based on a model proposed by Mani and Löwen^33^ as below:

1 $$f\left(n\right)=\frac{gn^{2}E_{r}\sigma }{d}-nE_{a}+\pi d\lambda$$

Here, the free energy (*f*(*n*)) of a cluster with *n* number of monomers is written as the sum of repulsive, attractive, and interfacial energies. The first term on the right-hand side of eq 1 accounts for the total repulsive energy given by the repulsive interparticle energy *E*_r_, individual monomer size σ, the size of the cluster *d*, the geometric factor *g*, related to the circular shape, and is of the order *n* ^2^, to account for long-range repulsive interaction. The second term is the total attractive energy given as a product of *n* and interparticle attractive energy *E*_a_. The last term accounts for the interfacial energy of the cluster given by the product of the cluster boundary length and the line tension λ. In a given 2D cluster, *n* and *d* are related as *n* = π*d* ^2^/4*a*, where a is the cross section of a particle given by *a* = πσ^2^/4. Substituting for σ and *d* in eq 1, the free energy of clustering for each particle is given as

2 $$\frac{f\left(n\right)}{n}=g\sqrt{n}E_{r}-E_{a}+2\lambda \sqrt{\frac{\pi a}{n}}$$

Minimizing eq 2 with respect to *n* yields the expression for the equilibrium cluster size n*, given as

3 $$n^{*}=\frac{\pi E_{a}}{gE_{r}}$$

Equation 3 gives the number of monomers in a stable cluster, which is proportional to the ratio of attractive to repulsive energies. We hypothesize that the repulsive interactions increase with the addition of water from the observations made using DLS. To verify this, we assume that the effect of addition of water may be encoded in the ratio $\frac{E_{a}}{E_{r}}$ as

$$\frac{E_{a}}{E_{r}}\propto {\left(\frac{1}{\phi_{w}}\right)}^{n}$$ 4

Since the size of a stable cluster is dependent on the number of monomers in a cluster, combining eqs 3 and 4, d* can be described as

$$d^{*}={\left(\frac{\alpha }{\phi_{w}}\right)}^{\beta }$$ 5

α and β are fitting parameters obtained by regression of the d* values measured at varying ϕ_w_. The details of the regression are provided in the SI. An *R* ^2^ value of 0.99 was estimated for the fitting, suggesting a good match with the experimental observation. Figure 3c shows the measured d* plotted against ϕ_w_, and the fitted curve is given as

6 $$d^{*}={\left(\frac{2.64}{\phi_{w}}\right)}^{3.78}$$

The strong dependence of the IONP cluster size on the amount of water added to the dispersion is indicated by the exponent β, obtained to be 3.78. This means that increased repulsive interparticle interactions lead to the breaking of IONP clusters. The increased repulsive interactions due to the addition of water could be attributed to the shrinkage of OA chains, possibly resulting in loosening of interparticle entangled chains. At sufficiently high ϕ_w_, water may penetrate through the OA layer to preferentially solvate the carboxy headgroup attached to the IONP surface,^34^ resulting in the development of charges and hence electrostatic repulsion.^24^ The loosely bound smaller clusters observed in Figures 3b and S2a–c indicate the effect of repulsive interactions as a result of the addition of water.

This effect was further pronounced when 0.1% (w/w) aqueous Tween 80 (T80) solution was added to the dispersion of IONPs in THF. Figure 3d shows time-dependent measurements for the systems at aqueous compositions (ϕ_aq_) of 0.5, 0.7, and 0.91. At a ϕ_aq_ of 0.5, IONP clusters of sizes close to 500 nm were measured through DLS. Interestingly, at a higher ϕ_aq_ of 0.7 and 0.91, the IONP clusters break into sizes closer to individual particle sizes; see the inset in Figure 3d. With an increased amount of T80 being added, the T80 molecules assemble at the interface of the OA and solvent mixture with the head groups pointing out. This provides steric stabilization to each particle at higher compositions, resulting in the disassembly of IONPs as reported earlier mediated by other surfactants.^35^ The breakage of IONP clusters into a well-dispersed system at an aqueous T80 composition of 0.91 was further confirmed using cryo-EM; see Figure 3e (also, see Figure S7). Individually dispersed IONPs stabilized by T80 at a composition of 0.91 could be seen in the micrograph, confirming the increase in repulsive steric interactions.

A side-by-side comparison between the sizes of ODE-, CP-, and ES-IONPs estimated from electron microscopy of dried particles and DLS of the systems with a ϕ_aq_ of 0.91 is presented in Figure 3f. The close match between the sizes obtained from both the techniques suggest that at a ϕ_aq_ of 0.91 and in the presence of T80, the particles are individually dispersed in the mixed solvent in all three systems.

### Effect of IONP Cluster Sizes on PLGA Encapsulation of IONPs

With the understanding of the colloidal behavior of IONPs in THF and mixed-solvent (THF–water) systems, we investigated the effect of the mixing ratio on their fate of PLGA encapsulation through FNP. We studied the encapsulation of the systems, which showed surfactant-mediated disassembly of IONPs in the mixed-solvent system at ϕ_*aq*_ of 0.5, 0.7, and 0.91. At first, the steady state of the reactor was determined by collecting samples at different intervals of time from the time of feed injection. Figure 4a shows the hydrodynamic diameter of PLGA nanoparticles (PNPs) obtained at various collection times (calculated from the time feeds were injected into the reactor) for an aqueous-to-organic flow rate (mL min^–1^) ratio (*Q*_a_:*Q*_o_) of 100:10 and 40:10. The results suggest that at a *Q*_a_:*Q*_o_ of 100:10, the sizes of the PNPs do not vary significantly over time. However, for 40:10, consistent sizes of PNPs were obtained after 30 s from the start of the process, indicating a longer transient time to achieve uniform mixing inside the micromixer. Nevertheless, in this study, a minimum equilibration time of 30 s was provided from the start of the process before samples were collected for characterization.

**Figure 4:**
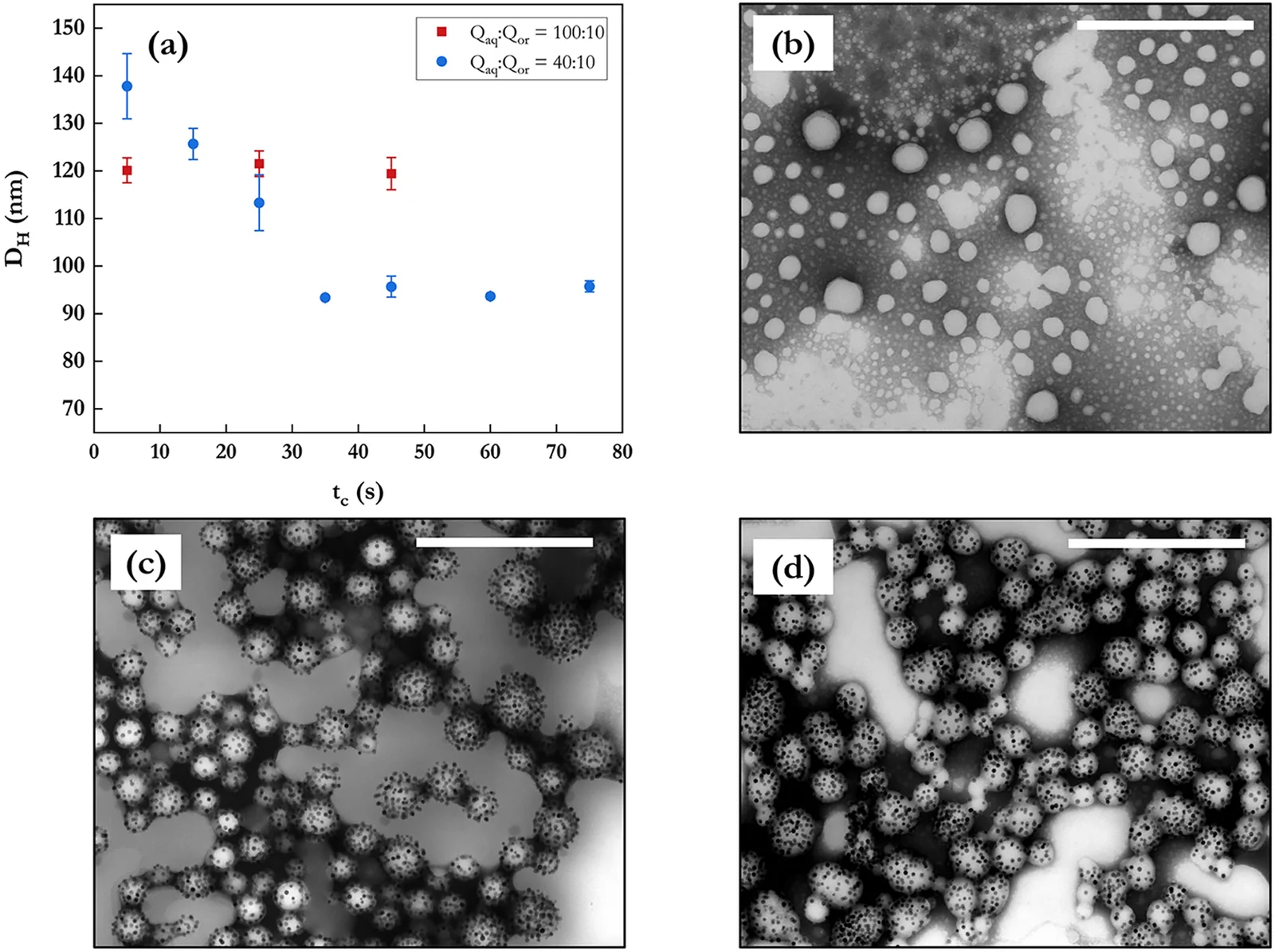
(a) *D*_H_ of PLGA nanoparticles obtained using FNP operated at mixing rates of 100:10 and 40:10, plotted against collection times (*t*_c_) from the start of the process. S­(T)­EM micrographs from encapsulation experiments performed using FNP with PLGA dissolved and IONPs dispersed in THF being injected with aqueous T80 solution at aqueous to organic mixing rates of (b) 50:50, (c) 70:30, and (d) 100:10. Scale bars are 500 nm.

The effect of the mixing ratio of the aqueous and organic streams on the encapsulation of IONPs was studied for *Q*_a_:*Q*_o_ of 50:50, 70:30, and 100:10. The aqueous stream comprised 0.1% T80 and the organic stream comprised 0.5 wt % PLGA and 3 mg mL^–1^ IONPs in THF. The IONP concentration in the final mixture was maintained constant for all mixing ratios. The products obtained were then purified as described in the Polymer Encapsulation of IONPs Using FNP section and imaged under S­(T)­EM for visualizing the encapsulation. Figure 4b–d shows the representative micrographs of the particles obtained at *Q*_a_:*Q*_o_ of 50:50, 70:30, and 100:10, respectively. The micrographs suggest that the mixing ratio of aqueous and organic streams had a direct influence on the fate of the encapsulation of IONPs. As discussed in the previous section, aqueous composition governs the colloidal behavior in a mixed-solvent system. In the presence of T80, we found that the IONP clusters break into smaller ones, making them well-dispersed at higher ϕ_w_. In line with these findings, at a *Q*_a_:*Q*_o_ of 50:50, no encapsulation was observed. For 70:30, however, the micrograph in Figure 4 shows partial encapsulation with many IONPs on the surface of the PLGA particles forming ’corona’-like assemblies. Furthermore, high encapsulation could be seen when a *Q*_a_:*Q*_o_ of 100:10 was used; see Figure 4d. These findings suggest that for effective encapsulation, IONPs need to exist in sizes less than 15 nm in the mixed-solvent environment during the course of encapsulation. Therefore, the understanding of the colloidal behavior of IONPs in the mixed-solvent environment becomes extremely crucial for effective encapsulation through FNP.

### Competing Factors Governing the Encapsulation of IONPs

Finally, we investigate the factors controlling the encapsulation of IONPs. We identified three main factors for this study: concentrations of PLGA and IONPs and the category of IONPs. A two-level full factorial experimental design was used to understand the effects of these three factors. The concentration levels of PLGA were 0.5 and 1.5 wt % and those of IONPs were 1 and 3 mg mL^–1^, which were assigned to ODE-, ES-, and CP-IONPs. The table for experimental design (ST1) is provided in the SI. The encapsulation experiments were performed using FNP at a fixed *Q*_a_:Q_o_ of 100:10, with the aqueous stream comprising 0.1 wt % T80. The particles formed from these experiments were then purified and analyzed through MP-AES to determine the iron:polymer weight ratio, defined as encapsulation efficiency here. The purified particles were also imaged using S­(T)­EM to visualize the fate of encapsulation.

The encapsulation efficiency and the corresponding S­(T)­EM micrograph for each experiment are provided in the SI (see Figures S9–S11). The S­(T)­EM micrographs of PLGA-encapsulated IONPs with high encapsulation efficiency within categories of IONPs are presented in Figure 5 for discussion. Figure 5a shows the PLGA encapsulation of CP-IONPs with an encapsulation efficiency of 97% formed using 0.5 wt % PLGA and 3 mg mL^–1^ IONPs. Under similar conditions, ODE- and ES-IONPs show 32 and 25% encapsulation, respectively, shown in Figure 5c,d. At a constant PLGA concentration of 0.5%, the encapsulation efficiency decreases from 97% to 62% when the IONP concentration was decreased from 3 to 1 mg mL^–1^, shown in Figure 5b. In line with these results, for each class of IONPs, the encapsulation efficiency gets affected in a stoichiometric manner depending on the IONP-to-polymer concentration ratio (*C*_i_:*C*_p_). A higher *C*_i_:*C*_p_ typically resulted in higher encapsulation efficiency. However, among the IONP categories, the CP-IONPs showed the highest encapsulation. Furthermore, it is interesting to note from the S­(T)­EM micrographs that the distribution of IONPs in the polymer particle matrix upon encapsulation also varies based on the type of IONPs. The CP-IONPs could be seen in patches inside the polymer particle (see Figure 5a,b), whereas the ODE-IONPs were found to be uniformly distributed inside polymer particles (see Figure 5c). The ES-IONPs, however, can be seen to be nonuniformly encapsulated inside a few polymer particles (see Figure 5d). We further discuss the role of surface coverage by the OA on the IONPs in governing the nature of encapsulation.

**Figure 5:**
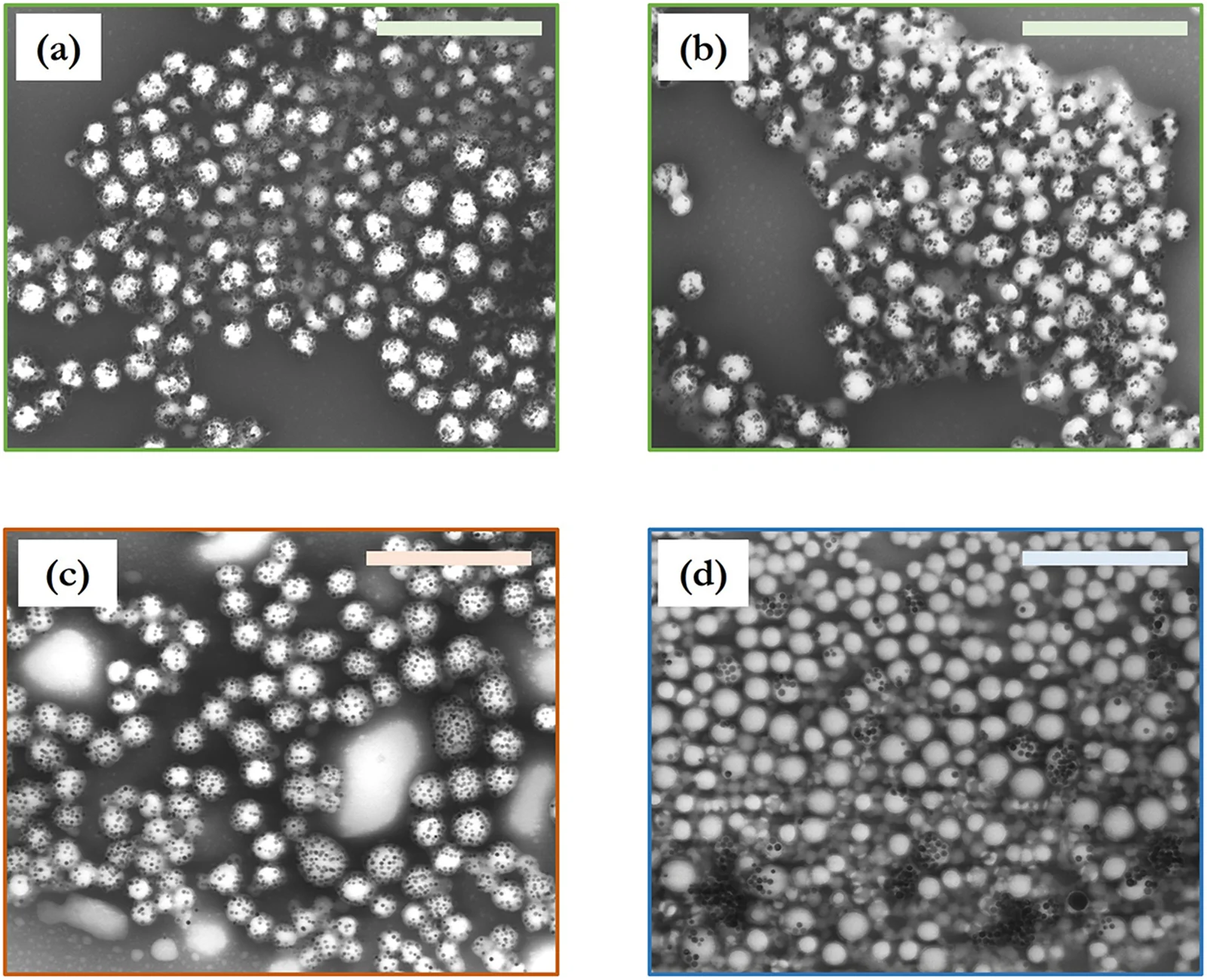
S­(T)­EM micrographs of PLGA-encapsulated IONPs synthesized using FNP. (a) and (b) P-IONPs made with CP-IONPs at concentrations of 1 and 3 mg mL^–1^, respectively. (c) P-IONPs made with ODE-IONPs at a concentration of 3 mg mL^–1^ and (d) P-IONPs made with ES-IONPs at a concentration of 3 mg mL^–1^. PLGA concentration in all the above systems was 0.5 wt %. Scale bars are 500 nm.

We define surface coverage (σ) as the mass of OA (*m*_OA_^p^) per unit surface area of particle of size *D*, given as

7 $$\sigma =\frac{m_{OA}^{p}}{\pi D^{2}}$$

For ODE-, ES-, and CP-IONPs, σ was estimated to be 8.5, 8.4, and 2.1 mg m^–2^, respectively. Details of the estimation of surface coverage are provided in the SI. It is interesting to note that the IONPs synthesized through thermal decomposition have similar surface coverage irrespective of their size differences, and the CP-IONPs have about 4 times lower surface coverage. The particles produced by thermal decomposition have well-defined morphologies and uniform OA coverage due to in situ functionalization during the synthesis.^36^ In contrast, the CP-IONPs have nonuniform morphology and lower OA coating due to ex situ functionalization. The particles tend to break into smaller clusters as water mixes with THF and T80 molecules start adsorbing onto the CP-IONP surface, observed in the controlled experiments discussed in the Effect of Water Addition on IONP Sizes section. Due to the uneven surface coverage of OA on the IONP surface, the particles are partially hydrophilic, which makes them accumulate near the interface of polymer-rich and -lean zones. These competing interparticle and particle–solvent interactions result in the IONPs being aggregated and entrapped inside and on the surface of the PLGA particle during the course of precipitation. Therefore, we observe high loading of CP-IONPs with aggregated patches inside and on the PLGA particle matrix (see Figure 5a,b).

The fate of encapsulation for ODE-IONPs, however, is different. The well-defined particle morphology and uniform OA surface coverage result in rapid stabilization due to steric repulsion upon mixing with the aqueous T80 stream. The encapsulated particles therefore form uniformly distributed ODE-IONPs inside the polymer particles, as shown in Figure 5c. Since the size of ES-IONPs is bigger (26 nm), even though they are stabilized and dispersed by adsorption of T80 due to similar surface chemistry as ODE-IONPs, the loading is lower and nonuniform mainly due to their sizes. Many ES-IONPs could be seen to have accumulated near the PLGA particle surface—Figure 5d. This observation is consistent with the one for PLGA-ODE-IONPs formed at a mixing rate of 70:30 as discussed in the Effect of IONP Cluster Sizes on PLGA Encapsulation of IONPs section, where the cluster sizes in the mixed solvent were ≈25 nm. These results indicate that there are two governing factors that significantly influence the fate of the encapsulation of IONPs: (i) the hydrophobicity of the particles determined by the uniformity and extent of surface coverage by OA and (ii) the cluster size of IONPs in the mixed-solvent environment that determines the nature of their entrapment in the PLGA particle matrix. This important understanding provides the next step for a complete mechanistic understanding of how these factors control encapsulation.

## Conclusions

Here, we have investigated the colloidal behavior of hydrophobic OA-IONPs synthesized by thermal decomposition and coprecipitation methods, resulting in two distinct surface coverage classes by OA and three size classes (11, 15, and 26 nm), in mixed aqueous–organic solvent systems. We probed the sizes of the IONPs over a range of solvent mixture compositions, starting from pure THF to water-rich mixtures using dynamic light scattering, and attempted to confirm the observations through cryo-EM imaging. The breakage of OA-IONP clusters upon addition of water was explained by a colloidal clustering model based on SALR interparticle potential. An empirically fitted power-law relationship was obtained between the equilibrium finite cluster sizes (d*) and the aqueous composition in the mixed solvent (ϕ_w_), encoded for the inverse of the attractive to repulsive interactions. The power law showed a strong dependence on ϕ_w_, suggesting that the addition of water induces repulsive interactions, which result in the breakage of larger clusters into smaller stable clusters. The effect was further pronounced when an aqueous solution of Tween 80 was added, resulting in well-dispersed particles at a ϕ_w_ of 0.91, also confirmed by cryo-EM. The effect of different cluster sizes on the nature of encapsulation was then studied by choosing three aqueous:organic mixing rates of 50:50, 70:30, and 100:10. The mixing ratio of 50:50 did not yield successful encapsulation, whereas at 70:30, IONPs were seen to form corona-like assemblies on the surface with some present inside the matrix of PLGA particles, with well-encapsulated systems obtained at 100:10 mixing rate, suggesting a direct relationship between the cluster sizes and the fate of encapsulation. Finally, we performed a systematic investigation to study the effect of PLGA and IONP concentrations on three size classes of OA-IONPs. In line with our previous reports, we found that the IONP:polymer ratio proportionally influences the encapsulation efficiency. However, the nature of the OA coating and the size influence both the distribution and extent of IONPs during encapsulation. The findings from this study can therefore be summarized as follows:

(1) The hydrophobic particles can exist in a range of cluster sizes when dispersed in solvents of mixed polarity, governed by competing interparticle attractive and repulsive interactions.
(2) The OA-IONPs get encapsulated in the PLGA particle matrix based on cluster sizes determined by the composition of organic and aqueous solvents during the course of encapsulation. In this work, we found that particles of sizes less than 15 nm, either well-dispersed or in clusters, result in high encapsulation. Therefore, the mixing rate ratio of organic and aqueous streams determines the extent of encapsulation.
(3) The extent of OA coating on the IONP surface determines the distribution of IONPs in the polymer particle matrix, governed by the partitioning of IONPs between the polymer-rich and -lean phases.

We believe that the above findings will contribute to understanding the colloidal behavior of hydrophobic particles, essential in the rational design of experiments for polymer encapsulation using flash nanoprecipitation.

## Supplementary Material



## Notes

Additional data and results pertaining to the calibration curves of MP-AES, oleic acid removal, calculation of encapsulation efficiency, model fitting for equilibrium cluster sizes, and experimental design to study the effect of polymer and IONP concentrations and surface coverage (PDF)
