# Supplementary material for: Competing Colloidal Interactions Govern Polymer Encapsulation of Hydrophobic Iron Oxide Nanoparticles
Source: Langmuir. 2026 Oct 8;42(41):30942–53. doi: 10.1021/acs.langmuir.6c04393 (PMC13689929; doi:10.1021/acs.langmuir.6c04393)
Supplement: Supplementary file 1 [file la6c04393_si_001.pdf]

Supporting Information

Competing colloidal interactions govern polymer encapsulation of hydrophobic iron oxide nanoparticles

Soumodeep Biswas[a,b,1], Takreem A. Sindhu[c,b,1], Muhammad Salman Haider[c], Ethayaraja Mani[a,d,e,f], Sulalit Bandyopadhyay[b,*]

email: sulalit.bandyopadhyay@ntnu.no

[a]Department of Chemical Engineering, Indian Institute of Technology Madras, Chennai, 600036, India

[b]Particle Engineering Center, Department of Chemical Engineering, Norwegian University of Science and Technology, Trondheim, 7491, Norway

[c]School of Chemical and Materials Engineering, National University of Science and Technology, Islamabad, 44000, Pakistan

[d]Centre of Excellence in Biodegradable Packaging, Department of Chemical Engineering, Indian Institute of Technology Madras, Chennai, 600036, India

[e]Centre for Soft and Biological Matter, Indian Institute of Technology Madras, Chennai, 600036, India

[f]School of Sustainability, Indian Institute of Technology Madras, Chennai, 600036, India

Number of pages	12
Number of figures	10
Number of schemes	1
Number of tables	1

Table of contents:

Sr. no.	Content	Page no.
1.	Calibration curves for elemental analysis using MP-AES	**S2**
2.	Purification of IONP dispersion through removal of excess oleic acid	**S3**
3.	Volume and number-based size distributions of IONPs in THF	**S4**
4.	Supporting Cryo-EM images	**S5**
5.	Linear regression for equilibrium cluster size model	**S6**
6.	Experimental design to study the effect of polymer and IONP	**S8**
7.	Results for the experimental design	**S9**
8.	Surface coverage by oleic acid on IONP surface	**S12**

1. Calibration curves for elemental analysis using MP-AES

For elemental analysis of iron oxide nanoparticles, standard solutions of iron (Fe) and gold were prepared at varying and identical concentrations. Au was added as internal standard for quantification of Fe to improve accuracy. The Fe/Au signal ratio makes MP-AES measurements more robust for elemental analysis.

$$\textbf{Calibration curve for Fe}: Intensity = 6802.646 * C_{Fe} - 0.049 \quad \text{(S1)}$$

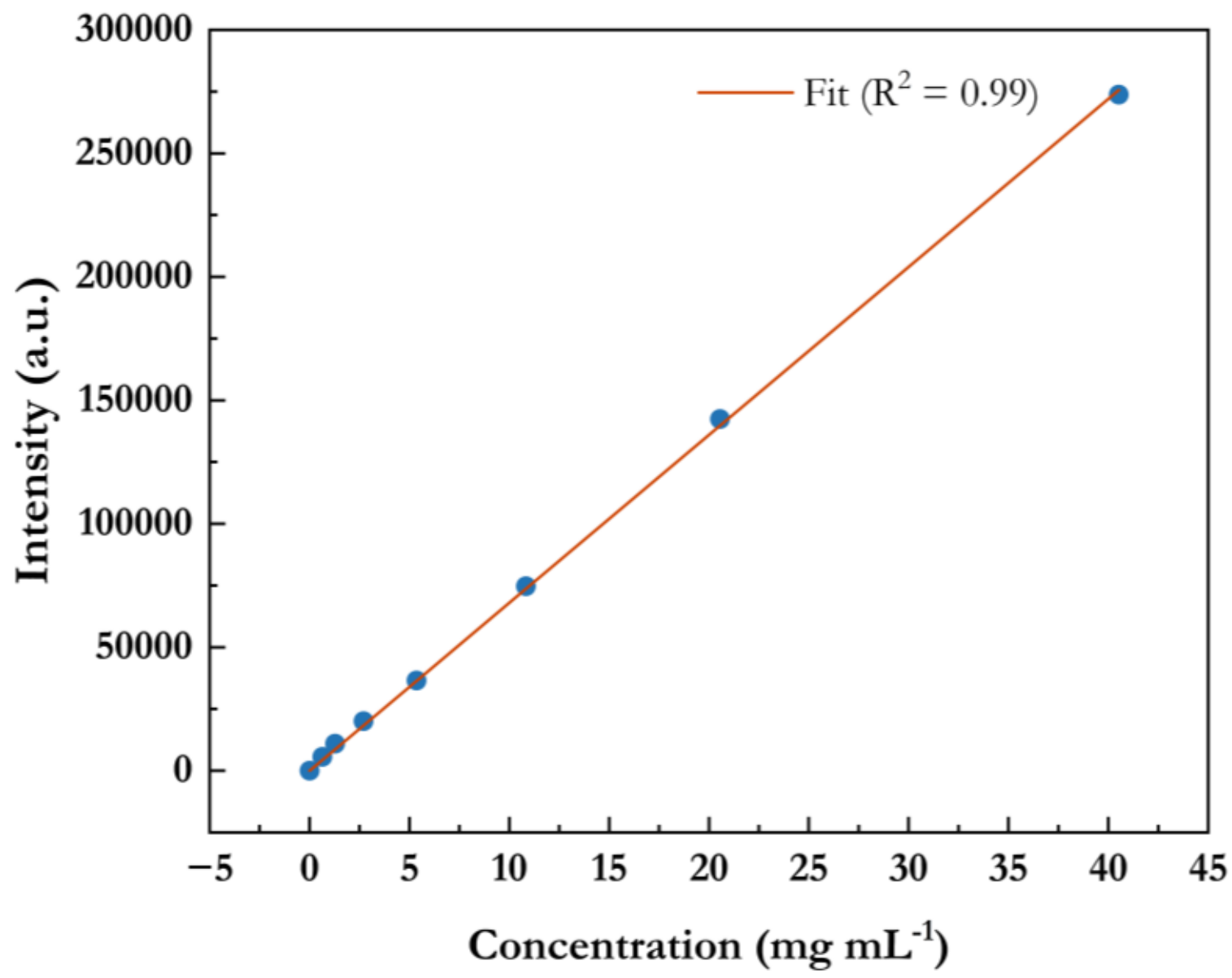

Figure S1: Plot showing calibration curve for quantification of Fe obtained using MP-AES for solutions of known concentrations

$$\textbf{Calibration curve for Au:} Intensity = 7103.067 * C_{Au} - 0.273 \quad \text{(S2)}$$

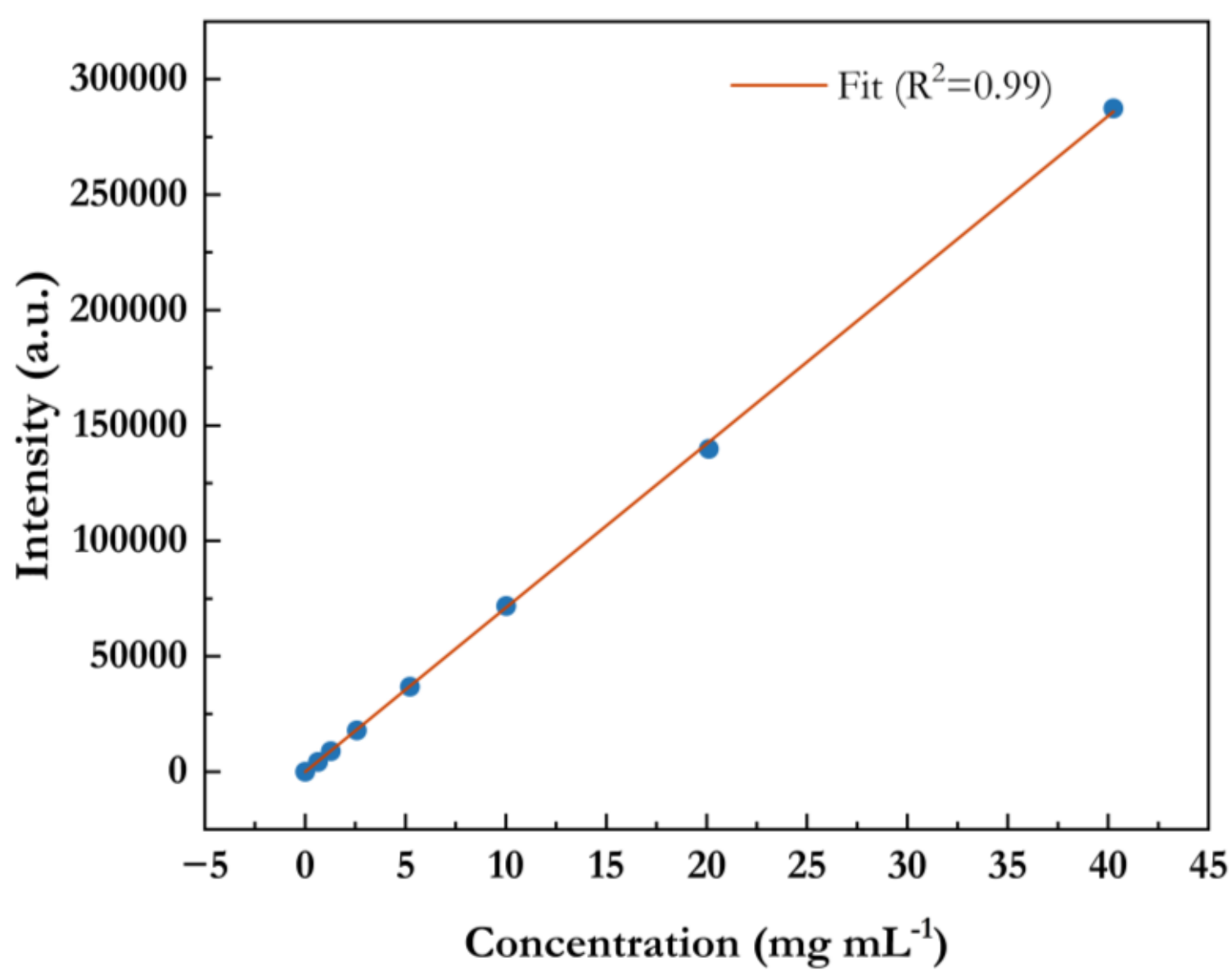

Figure S2: Plot showing calibration curve quantification of Fe obtained using MP-AES for known concentrations.

2. Purification of IONP dispersion through removal of excess oleic acid:

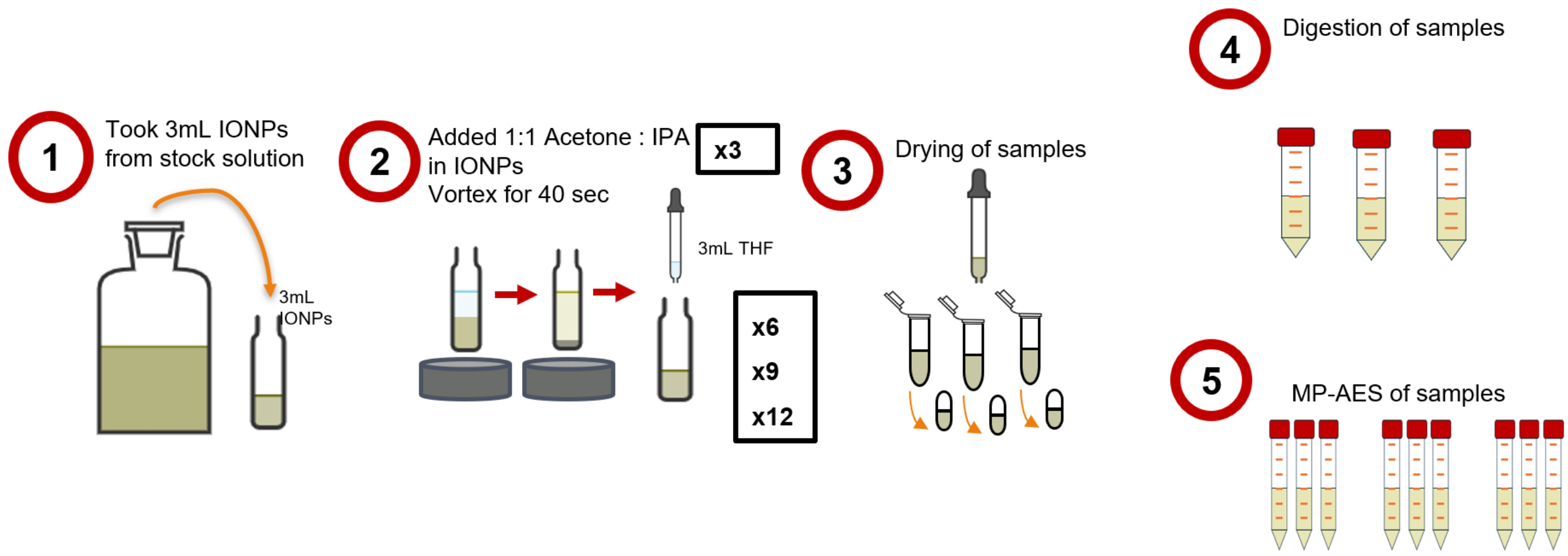

Figure S3: Schematic of the procedure followed for estimating the oleic acid removal from IONPs when subjected to multiple wash cycles

Sample calculation for oleic acid content for ODE-IONPs after three wash-cycles:

For a sample volume of 1 mL, the IONPs were dried using block heater for 24 hrs. The mass of dried IONPs (M_{NP}), comprising of both IONPs and oleic acid, were found to be, $M_{NP} =$ 15.52 mg. Post, elemental analysis, the iron oxide content was estimated using the calibration curve.

Estimated iron oxide content: $C_{Fe_xO_y} = 13.73$ mg mL^{-1}

Therefore, mass of iron oxide: $M_{Fe_xO_y} = 1.73$ mg

And, mass of oleic acid: $M_{OA} = 13.79$ mg

The relative amount of oleic acid in the sample is thus, estimated as:

$$\%OA = \frac{M_{OA}}{M_{NP}} 100 \qquad (S3)$$

$$= 88.85\ \%$$

3. Volume and number-based size distributions of IONPs in THF

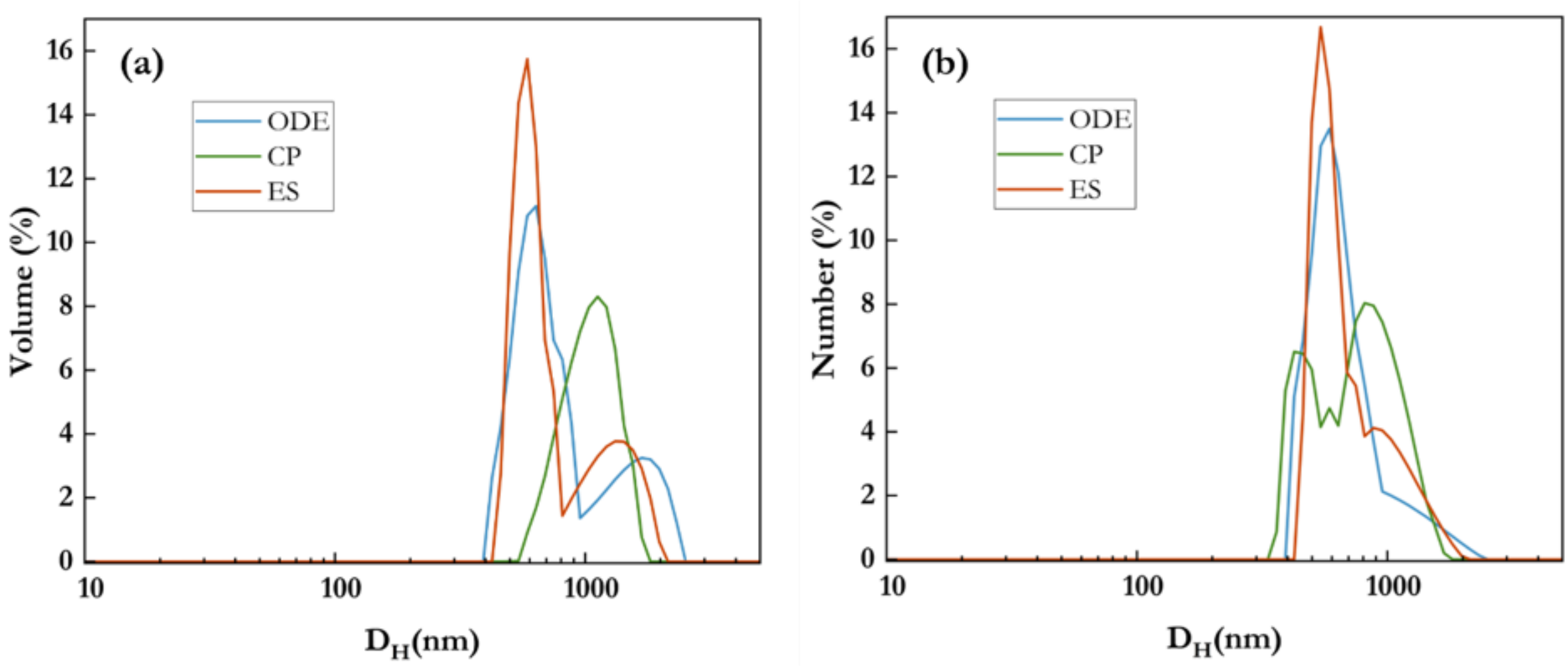

Figure S4: (a) Volume and (b) number weighted size distributions for ODE-, ES- and CP- IONPs when dispersed in THF, obtained using DLS.

4. Supporting Cryo-EM images

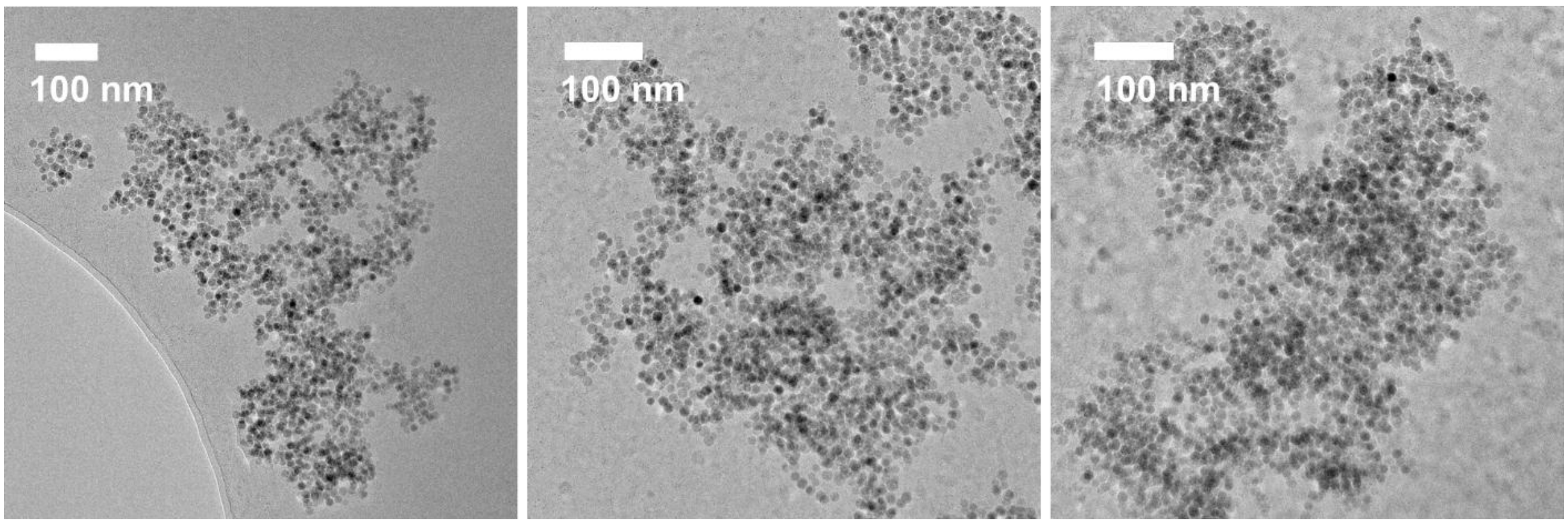

Figure S5: Cryo-EM images of IONPs dispersed in THF

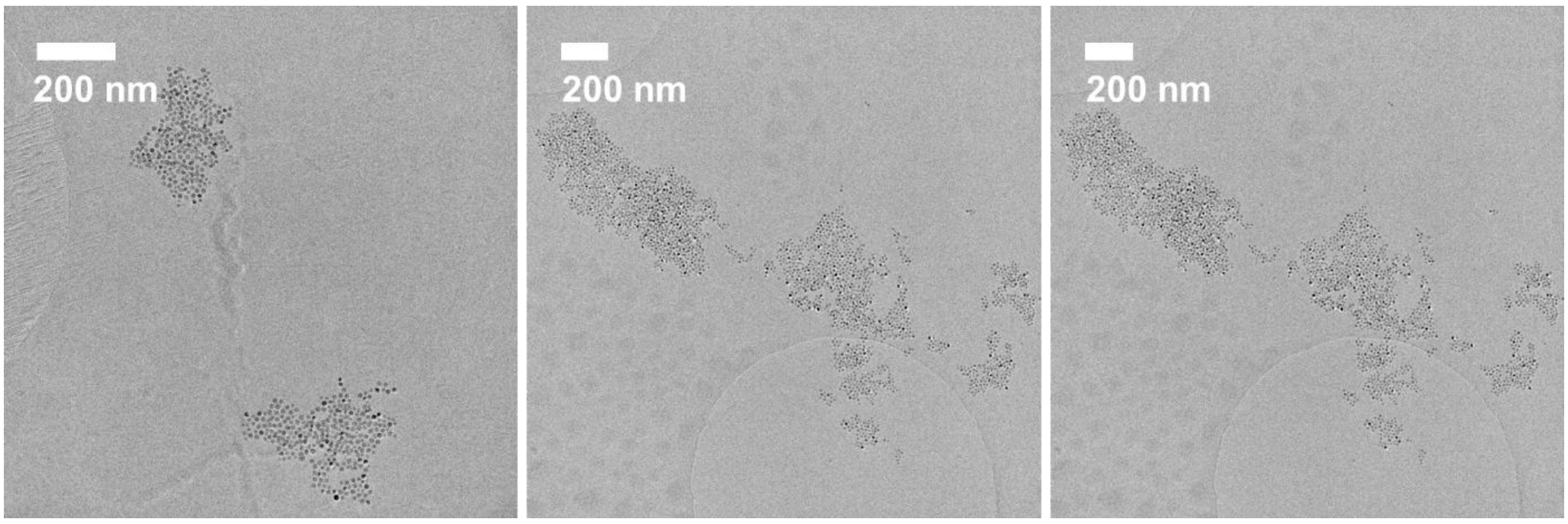

Figure S6: Cryo-EM images of IONPs dispersed in THF-water mixture at 0.9:9.1 volume ratio

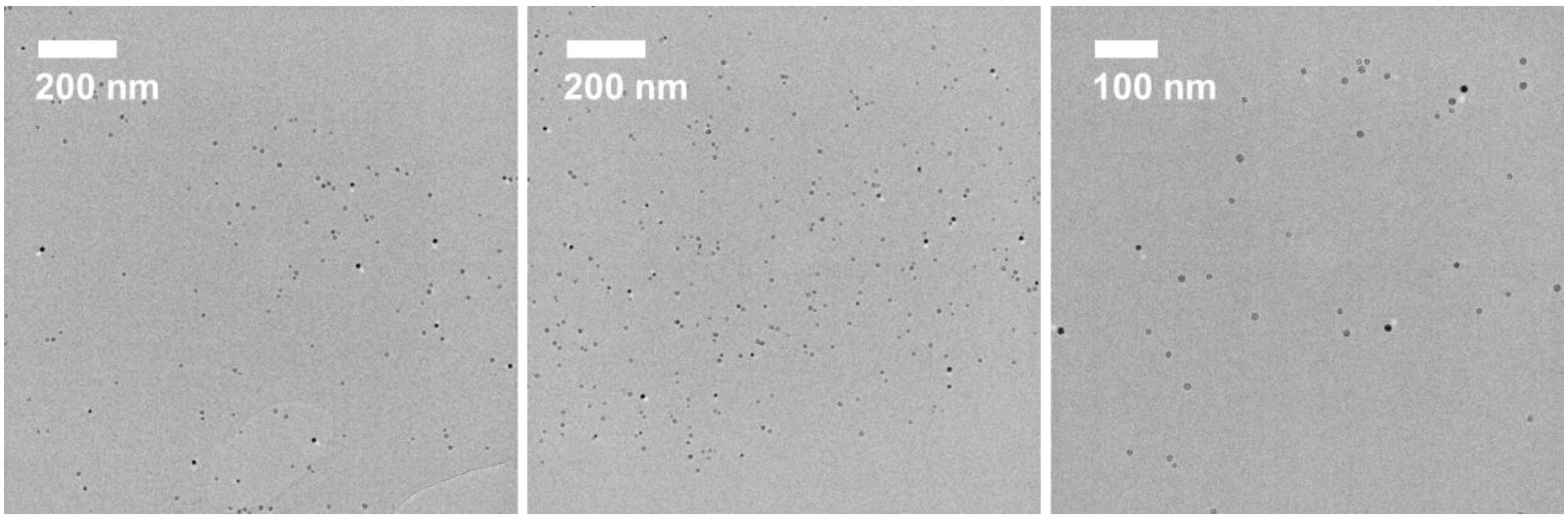

Figure S7: Cryo-EM images of IONPs dispersed in THF-aqueous tween80 solution (0.1 wt%) mixture at 0.9:9.1 volume ratio

5. Linear regression for equilibrium cluster size model

The expression derived from the equilibrium clustering model to describe a relationship between the finite cluster size and the composition of water in the solvent mixture is given as ((7) in the main text):

$$d^* = \left(\frac{\alpha}{\phi_w}\right)^{\beta} \tag{S4}$$

Here, α and β were obtained using regression. The above expression can be linearized by logarithmic treatment as follows:

$$\ln(d^*) = \beta \ln(\alpha) - \beta \ln(\phi_w) \tag{S5}$$

Let, $\beta \ln(\alpha) = A$, and $-\beta = B$. Therefore, the linear expression is given as,

$$\ln(d^*) = A + B \ln(\phi_w) \tag{S6}$$

Linear regression was performed on the above expression to obtain the following result;

$$\ln(d^*) = 3.67 - 3.78 \ln(\phi_w) \tag{S7}$$

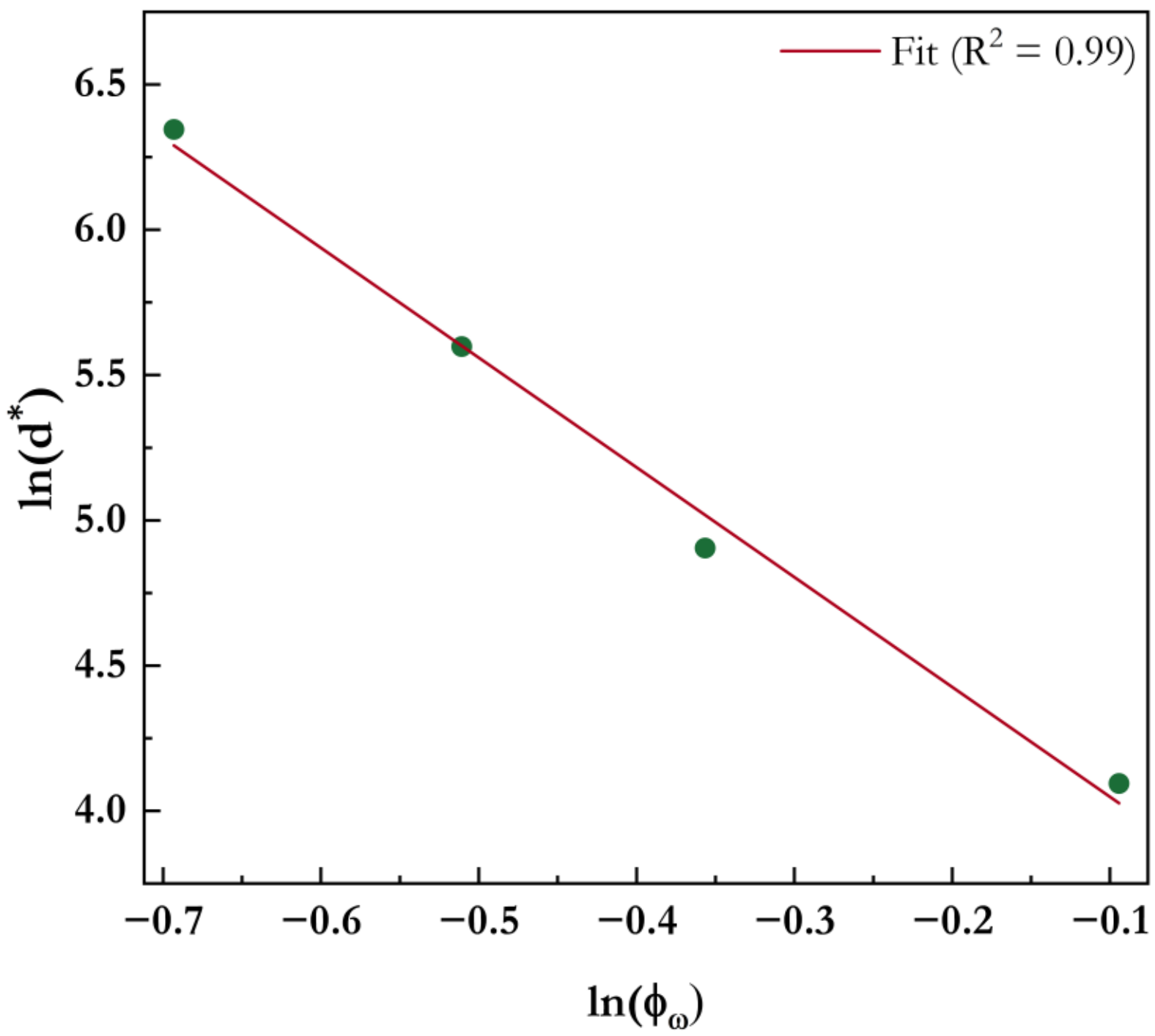

Figure S8: Linear regression of equation (3) based on the experimentally measured sizes of IONPs at varying ϕ_w

Therefore, (3) can be written as:

$$\ln(d^*) = 3.67 - 3.78\ln(\phi_w) \tag{S8}$$

Which yields, $\alpha = 2.64$ and $\beta = 3.78$. The relationship between equilibrium cluster size and the composition of water in the mixture of solvent is given as:

$$d^* = \left(\frac{2.64}{\phi_w}\right)^{3.78} \tag{S9}$$

6. Experimental design to study the effect of polymer and IONP concentration on encapsulation

Table ST1: Experimental design and conditions for studying the effect of PLGA and IONP concentrations for all three categories of IONPs on encapsulation efficiency

Sr. No.	Category of IONPs	Design		Measured		
		Concentration of PLGA (wt%)	Concentration of IONPs (mg mL^{-1})	Concentration of PLGA (wt%)	Concentration of IONPs (mg mL^{-1})	(IONPs in THF) particle/mL
1	ODE-IONPs	0.5	1	0.56	0.99	1.1E+14
2		0.5	3	0.71	3.02	3.4E+14
3		1.5	1	1.68	1.04	1.2E+14
4		1.5	3	1.99	3.05	3.5E+14
5	ES-IONPs	0.5	1	0.54	1.02	1.6E+13
6		0.5	3	0.63	3.12	4.9E+13
7		1.5	1	1.64	1.00	1.6E13
8		1.5	3	1.86	3.02	4.7E+13
9	CP-IONPs	0.5	1	0.56	1.24	1.2E+13
10		0.5	3	0.64	3.51	3.5E+13
11		1.5	1	1.59	1.14	1.1E+13
12		1.5	3	1.74	3.51	3.4E+13

7. Results for the experimental design

Estimation of encapsulation efficiency:

Eg: For the PIONPs using ODE-IONPs at 0.5% PLGA and 1mg/mL of IONPs, 5mL of sample was dried on block heater at 99ºC for 24 hours that contains polymer, IONPs and oleic acid. The dried mass of sample was $M_{PIONPs} = 0.35\ mg$

The dried mass is then dissolved in a digestate and diluted to detect the concentration in the range of calibration. Once, the concentration of iron oxide ($C_{Fe_xO_y}$) in the digestate is obtained, the mass in the mass of iron oxide is then scaled back to the sample volume as (α: gold correction factor, DF: Total dilution factor):

$$M_{Fe_xO_y} = \quad C_{Fe_xO_y} \,.\, \alpha \,.\, DF \qquad \text{(S10)}$$

$$= 0.02\ mg$$

From the previous analysis of IONPs, we know the concentration of oleic acid present (0.03 mg), Therefore the mass of polymer excluding the OA-IONP:

$$M_P = M_{PIONPs} - M_{Fe_xO_y} - M_{OA} \qquad \text{(S11)}$$

$$= 0.30\ mg$$

Therefore, the encapsulation efficiency (EE) is given as:

$$EE\ \% = \frac{M_{Fe_xO_y}}{M_P}\ 100 = 6.67\% \qquad \text{(S12)}$$

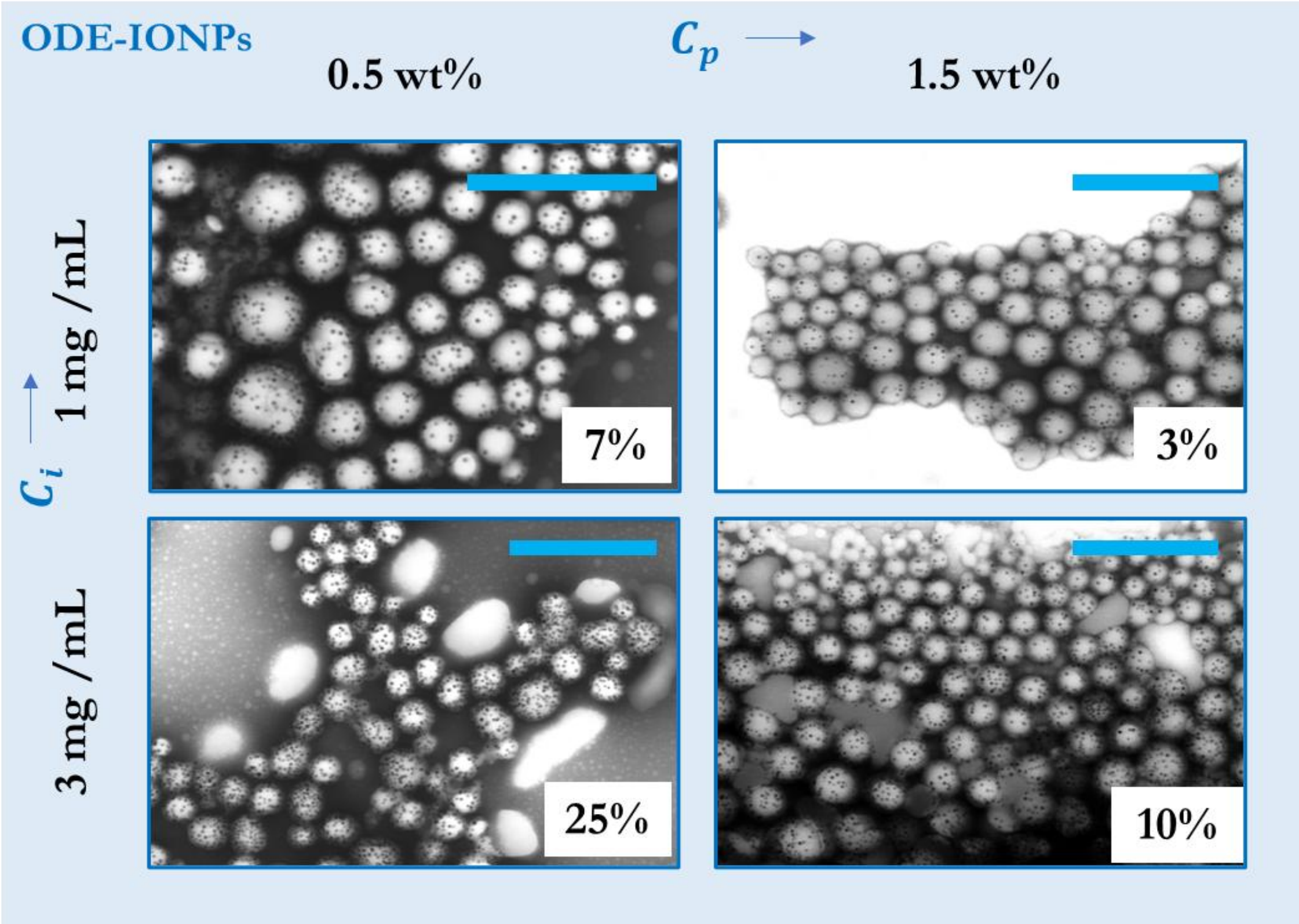

Figure S9: S(T)EM images of P-IONPs synthesized with ODE-IONPs at given polymer (C_i) and IONP (C_p) concentrations. Scale bars are of 500 nm.

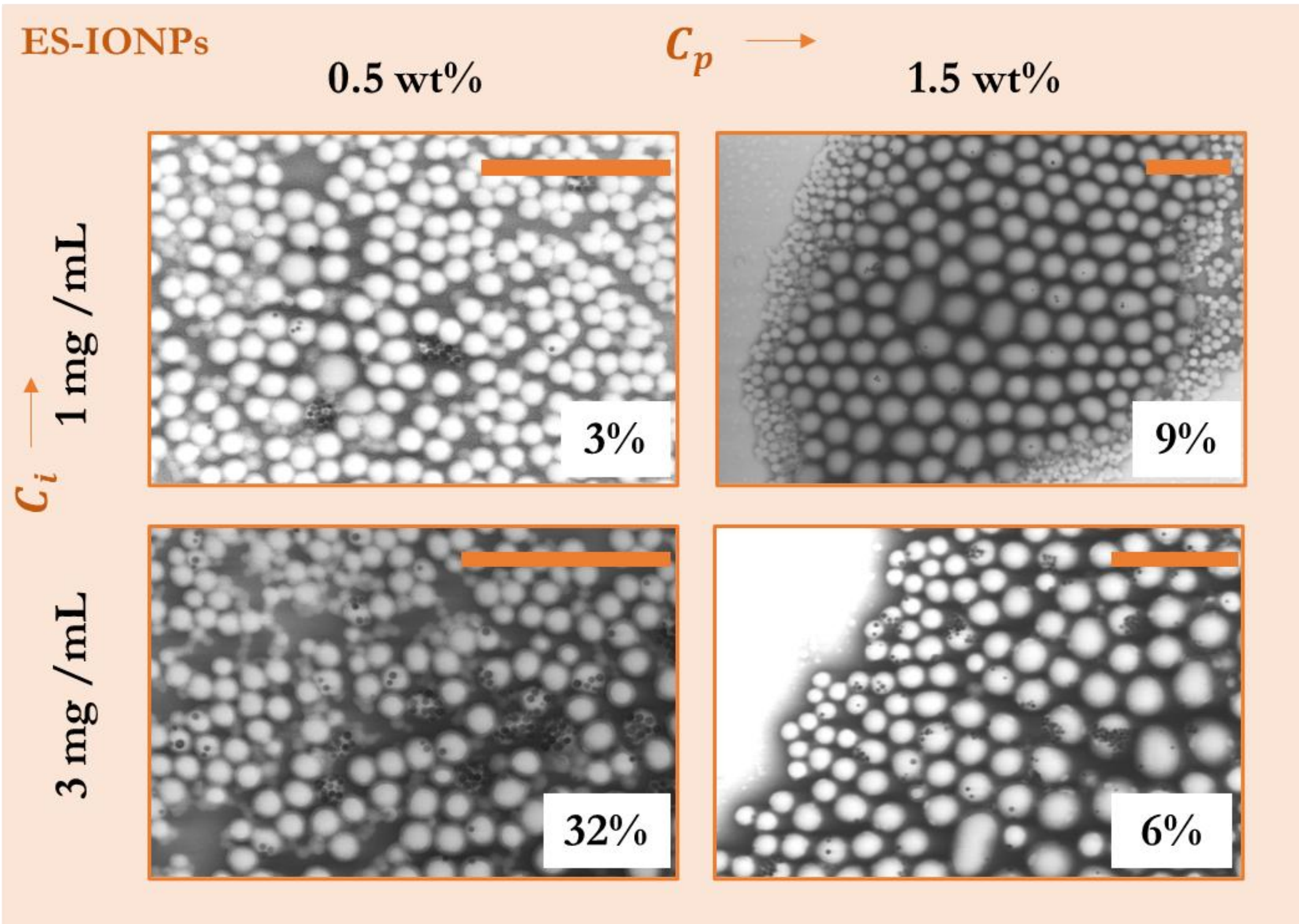

Figure S10: S(T)EM images of P-IONPs synthesized with ES-IONPs at given polymer C_i and C_p. Scale bars are of 500 nm.

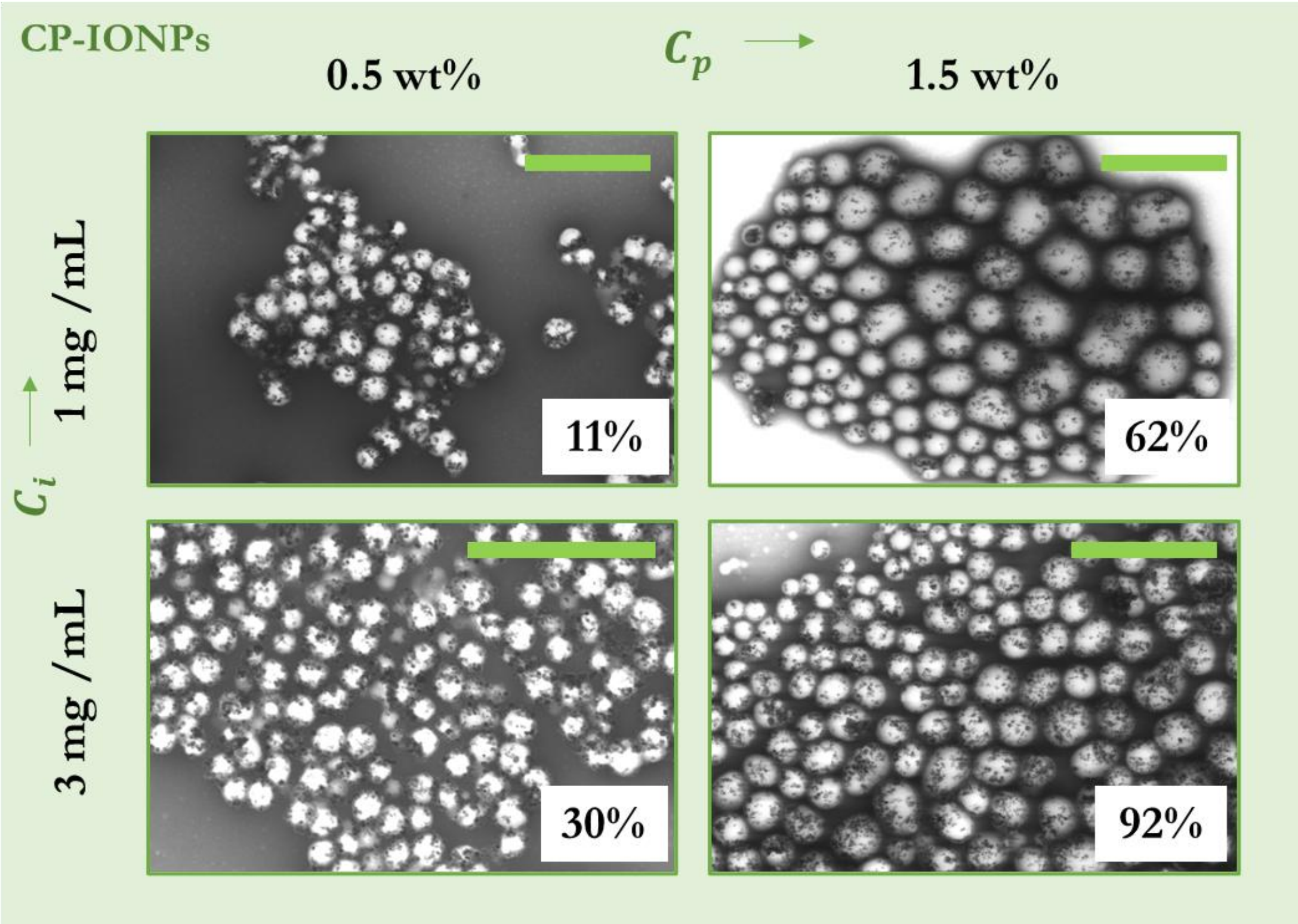

Figure S11: S(T)EM images of P-IONPs synthesized with CP-IONPs at given polymer C_i and C_p. Scale bars are of 500 nm.

8. Surface coverage by oleic acid on IONP surface

Surface coverage (σ) on a spherical particle of diameter D is defined as:

$$\sigma = \frac{M_{OA}^{P}}{\pi D^2} \tag{S13}$$

Where, M_{OA}^{P} is the mass of oleic acid per particle, defined as:

$$M_{OA}^{P} = \frac{M_{OA}}{\#\ of\ particles} \tag{S14}$$

The number (#) of particles are calculated by using the estimation of total iron oxide content from the elemental analysis as described before.

$$\# \ of \ particles = \frac{M_{FexOy}}{\rho_{FexOy}\frac{\pi D^3}{6}} \tag{S15}$$

Here, ρ_{FexOy} is the density of iron oxide.
